# Gene Expression Depends on the Interplay Among Growth, Resource Biogenesis, and Nutrient Quality

**DOI:** 10.1021/acssynbio.4c00828

**Published:** 2025-05-29

**Authors:** Juhyun Kim, Alexander P.S. Darlington, Said Muñoz-Montero, Rafael Montenegro, Perrine Dalby, Noemí Herrera-Martín, Alice Banks, Satya Prakash, Karen Polizzi, Declan G. Bates, José I. Jiménez

**Affiliations:** † School of Life Science, BK21 FOUR KNU Creative BioResearch Group, 34986 Kyungpook National University, Daegu 41566, Republic of Korea; ‡ School of Engineering, University of Warwick, Coventry CV4 7AL, United Kingdom; § Department of Life Sciences, 4615 Imperial College London, South Kensington Campus, London SW7 2AZ, United Kingdom; ∥ Department of Chemical Engineering, 4615 Imperial College London, South Kensington Campus, London SW7 2AZ, United Kingdom

**Keywords:** ribosomes, cellular economics, metabolic constraints, (p)ppGpp, resource allocation, metabolic burden

## Abstract

The gene expression capacity of bacteria depends on the interplay between growth and the availability of the transcriptional and translational machinery. Growth rate is accepted as the physiological parameter controlling the allocation of cellular resources. Understanding the relationship between growth and resources is key for the efficient design of genetic constructs, but it is obscured by the mutual dependence between growth and gene expression. In this work, we investigate the contributions of molecular factors, growth rate, and metabolism to gene expression by investigating the behavior of bacterial cells grown in chemostats. Using a model of the whole cell and validating it experimentally, our results show that while growth rate and molecular factors, such as the number of rRNA operons, set the abundance of transcriptional and translational machinery, it is metabolism that governs the usage of resources by tuning elongation rates. We show, using a biotechnologically relevant example, that gene expression capacity can be maximized using low growth in a high-quality medium. These findings unveil fundamental trade-offs in physiology that will inform future bioprocess optimization.

## Graphical abstract

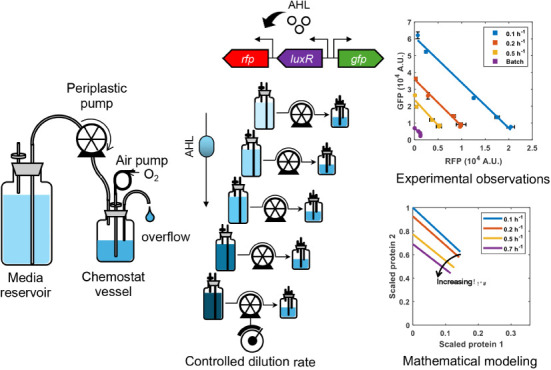

## Introduction

The ultimate goal of synthetic biology is to engineer reliable and robust genetic circuits. These circuits have a range of potential applications in biomedicine, industry and environmental science,^1^ but ensuring their reliable performance requires precise control over gene expression. To this end, it is crucial to understand the inherent constraints on gene expression created by the limited cellular economy, in order to allow optimal allocation of resources between host and circuit genes. Recently, researchers have observed that the expression of a particular gene can affect the activity of another seemingly unconnected gene due to sharing of the cell’s resources.^2–4^ In bacteria, such resource-coupled gene expression is mainly caused by limitations in the numbers of ribosomes.^5–11^ Several approaches have been developed to reduce this coupling,^7,9,11,12^ for instance, we successfully mitigated competition for ribosomes in coexpressed genes using quasi-orthogonal ribosomes combined with a resource allocation controller.^12^

The association between cellular growth and ribosome abundance has previously been investigated in order to understand how cells allocate their protein synthesis capacity. As the synthesis of ribosomes is energetically costly, cells need to maintain an optimal concentration of these macromolecules to maximize cellular fitness.^13,14^ In bacteria, evolutionary trade-offs have shaped the number of rRNA operons to achieve optimal ribosomal biosynthesis. For instance, wild type Escherichia coli carry seven copies of the rRNA operons, and adapt to nutritional perturbations more quickly that other isogenic strains with different copy numbers of these operons.^15^ The cellular concentration of ribosomes is also controlled by the nutritional stress-induced alarmone (p)­ppGpp.^16–20^ As a response to amino acid starvation (the stringent response), cells accumulate (p)­ppGpp, which represses the expression of both rRNAs and ribosomal-protein coding genes through direct interaction with the RNA polymerase.^21^

Bacterial growth rate acts as a global regulator of ribosomal concentrations by coordinating the distribution of the cellular proteome. This allocation has been described by partitioning the E. coli proteome into three fractions: the R fraction (ribosomes/ribosome-associated), the E fraction (containing metabolic enzymes), and a growth independent fraction.^22–24^ Cells allocate more of their proteome to ribosome-associated fractions under nutrient rich conditions to support maximum growth, and thus lower amounts of metabolic proteins are produced. In contrast, under poor nutrient conditions, cells synthesize more metabolic proteins to produce ATP at the expense of ribosome production, which leads to slower growth.^22–24^ Consequently, the synthesis of ribosome-associated proteins competes with the production of nonribosomal proteins.

To investigate such cellular fitness strategies, growth rate (and hence ribosome biosynthesis) has been manipulated by altering nutrient quality.^25–27^ However, this can potentially produce confounding results, due to the impact of different metabolic effects on the resource allocation. For example, different carbon sources could lead to distinct profiles of amino acid synthesis which in turn could affect translation beyond the availability of ribosomes leading to a different translational capacity, which in this work we define as the product of the peptide elongation and number of ribosomes. In addition, the impact of growth rate on such resource-mediated coupling has so far not been explored, and this question is crucial to ensuring that multiple genes are expressed at the right combination and concentration under dynamic resource allocation regimes.

To address these challenges, we investigate resource allocation in the cellular proteome by using a continuous culturing system that enables a systematic investigation of different growth scenarios for the same metabolic conditions. By combining theory and experiments, we obtain novel insights into the interplay between transcription and translation and the role of rRNA operons. These insights allow us to untangle the relationship between growth and metabolism to identify the impact on gene couplings of both carbon sources and the (p)­ppGpp network. These results shed new light on fundamental trade-offs in microbial physiology and will enable optimized cultivation of microbial cell factories for diverse biotechnological applications, as exemplified here by maximizing the biosynthesis of violacein.

## Results

### Growth Rates Set Gene Expression Trade-Offs in Carbon-Rich Conditions

It is well established that changing nutrients within a growth medium impacts both cell growth rate and ribosome content, with both being correlated.^27–29^ Moreover, the interplay between gene expression and growth on a range of media has previously been established.^25–27^ However, in these experiments the changes to the cell’s metabolism (such as changing carbon source) cause concurrent changes to growth rate, resource biosynthesis and gene expression. To dissect these relationships independently we utilized a chemostat to allow for the tight control of bacterial growth in glucose conditions by changing the dilution rates in the reactor. This allows the manipulation of bacterial growth rates and the quantification of gene expression levels under identical external nutrient conditions. Under these conditions we determined the intracellular concentration of DNA, RNA and proteins in E. coli MG1655 at the following growth rates: 0.1, 0.3, and 0.5 h^–1^, as determined by the dilution rate of the minichemostat (Figure 1).

**Figure 1:**
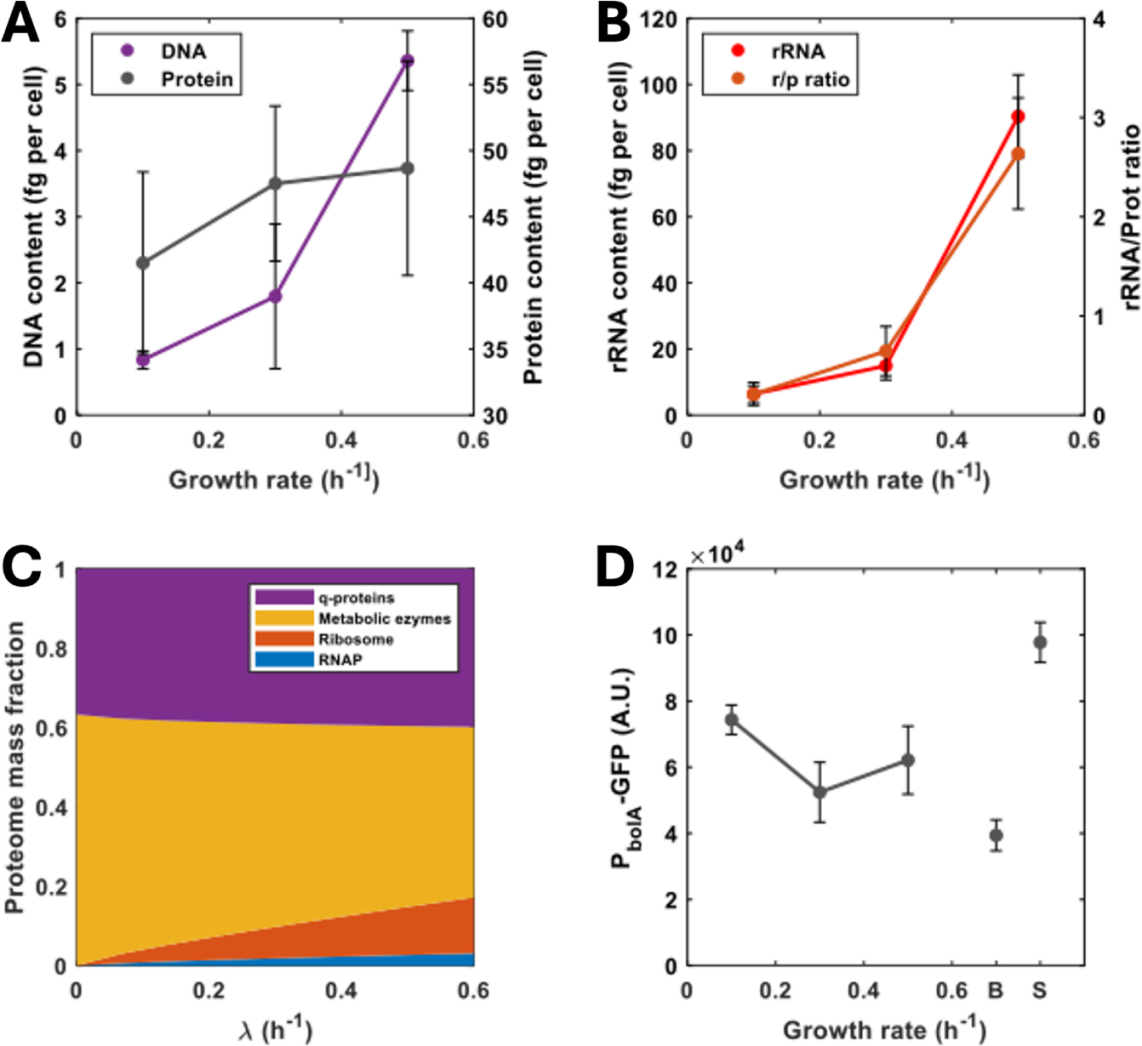
Impact of growth rates in the chemostat on macromolecular cellular components. Cells were cultured in the chemostat at different growth rates and processed to determine their macromolecular components as described in the methods section. (A) The cellular DNA content increases with the growth rate in the chemostat, while total protein content remains constant. (B) The rRNA levels (proxy for ribosomal content) present in the total RNA extracted from the cells also increase with the growth rate and, as a consequence, so does the rRNA/protein ratio typically used to characterize resource allocation strategies. (C) Our model captures the experimental observations in the chemostat. The model was built and fit to existing batch culture data as described in the Methods. The model predicts small changes in total protein content and increased ribosomal synthesis. (D) Cellular stress, monitored as the expression levels of *PbolA-GFP* fusion does not show significant changes across growth rates in the chemostat, suggesting that macromolecular changes are largely independent of stress responses. For comparison, results from the same reporter strain grown in batch culture at mid exponential (B) and stationary phase (S) are shown. Bars represent the mean ± 1 SD *N* = 3.

Our results verify that the chemostat recapitulates previous observations^22^ showing that the DNA and RNA contents and RNA/protein ratios correlate (Pearson’s *R* > 0.85; *p* < 0.05) with the growth rate of the culture (Figure 1A,B,D). In a chemostat, high dilution rates resemble the conditions of the exponential phase of batch cultures normally used to assess intracellular components.

By combining mechanistic models of gene expression and natural feedback with phenomenological models of growth and a simple representation of metabolism, we produced a tractable model that can recapitulate the different distributions of cellular proteins. For a full description of the equations of the model see the Methods. The model goes beyond existing ones,^12,30,31^ by combining a simple representation of metabolism with (p)­ppGpp based regulation and a mechanistic, rather than phenomenological, model of transcription and ribosome biogenesis. Our complete model accounts for the key universal bacterial constraints: (i) finite biosynthetic capacity (e.g., protein production capacity), (ii) competition for total number of proteins, (iii) competition for RNA polymerases by mRNA and rRNA promoters (a new addition), (iv) competition for ribosomes by mRNAs, (v) resource biosynthesis (both production of RNA polymerase and ribosomes, including their autocatalysis), and (vi) dynamic growth-based feedback.

A schematic of the full model is shown in Figure 2 and full mathematical details are described in the Methods. The model considers the growth of cells in the presence of a single growth-limiting external substrate (*S*) which is imported into the cell and converted by enzymes (*p*_*E*_) to produce a pool of internal metabolites *M*_*i*_ representing amino acids, nucleotides, ATP and NADH etc. Concurrently tRNAs (*t*_*u*_) are produced in proportion to ribosome biogenesis and are charged in a biomolecular reaction which is catalyzed by enzyme *p*_*E*_ and consumes *M*_*i*_. The gene expression dynamics of enzymes, ribosomes and host biomass proteins (the Q-fraction described by^22^ are captured by a simple two state model for each gene which captures the dynamics of the following processes: First, the RNA polymerase (*P*) reversibly binds to free promoters to form transcription complexes ( ${\mathrm{k̃}}_{j}$ , where *j* is the gene of interest). These are “consumed” by transcription *T*_*X*_ (·) to produce mRNAs (*m*_*j*_). The mRNA are reversibly bound by functional ribosomes (*R*) to produce the translation complex $\left({\mathrm{c̃}}_{j}\right)$ ). The mRNA are released upon termination of translation at rate *T*_*L*_ (·), which produces new proteins (*p*_*j*). By applying the quasi-steady state approximation to this scheme (similar to that in refs 4,30 ), we track only the mRNA and protein dynamics with intermediate species assumed to be at quasi-steady state. We assume that the transcription process is dependent upon the cell’s internal energy state (*M*) but does not consume metabolites, as previous works have shown most cellular energy consumption is by translation (see ref 31). Our translation function is dependent upon the concentration of charged tRNAs (*t*_*c*_) which are consumed and release uncharged tRNAs (*t*_*u*_). We modeled gene expression regulation by scaling the RNA polymerase-promoter association rate through a phenomenological description of the action of ppGpp, using the function θ which is effectively the inverse of pGpp concentration and is proportional to the charged/uncharged tRNA ratio^32^ (see Methods, Figure 2). The dynamics of resource biosynthesis mirrors those of other genes with ribosome biosynthesis modeled as a two-step process consisting of transcription of rRNAs and transcription/translation of ribosomal proteins. These bind to production functional ribosomes. Growth rate is calculated dynamically based on the global peptide production rate (i.e., the product of translational elongation rate γ­(*t*_*c*_) and the sum of the mRNA-bound translational complexes $\sum \left(\overset{\sim }{c_{j}\;}\right)$ ) as in previous works.^12,30,31,33^ The model was fit to the resource economy data from refs 34 and 22 as described in the Methods (Figure S1).

**Figure 2:**
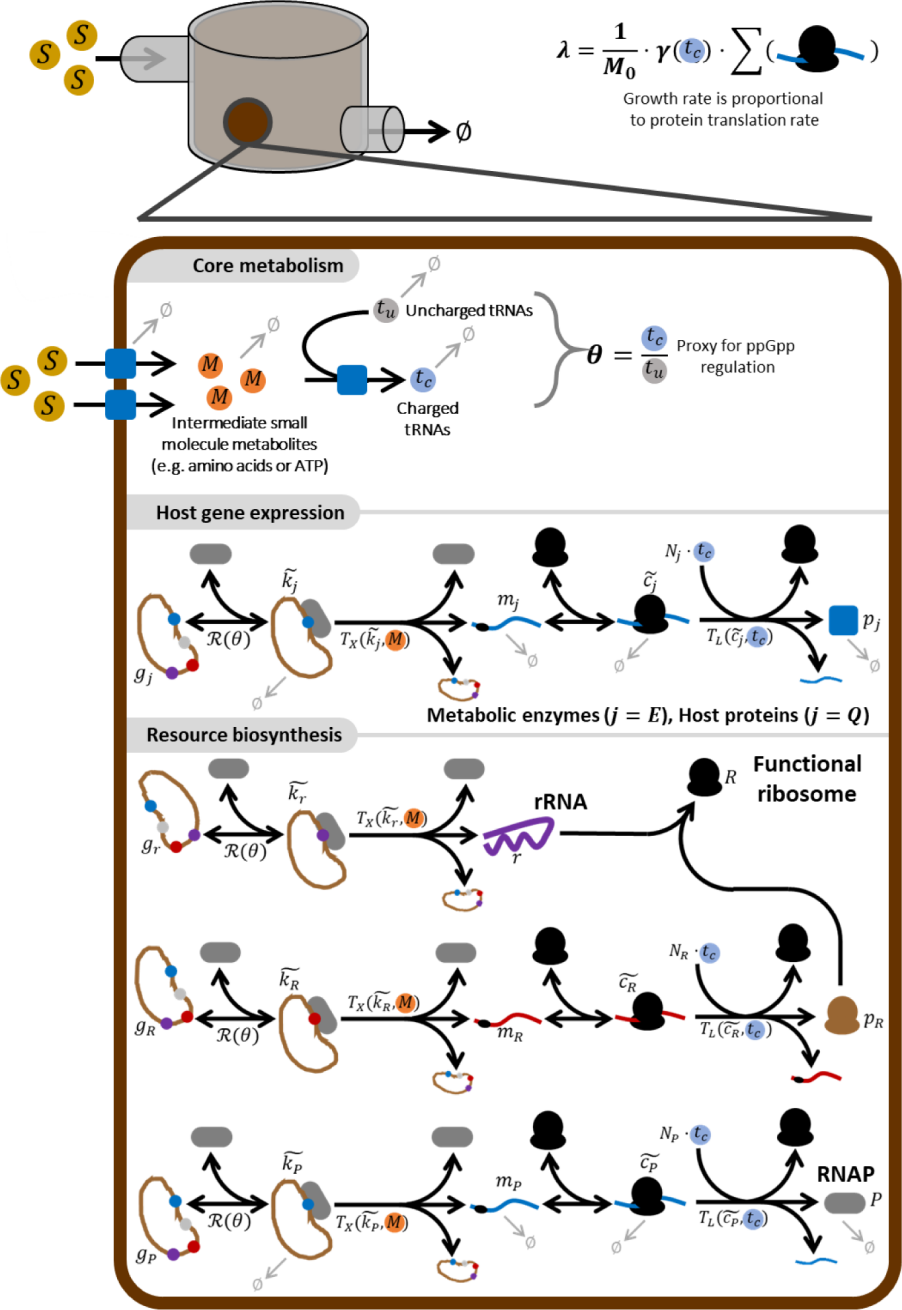
Ordinary differential equation model describes a simple cell with a four-state metabolism, gene expression and resource biosynthesis. The external nutrient (*S)* is imported and metabolized by enzyme proteins (*p*_*ε*_) to become the internal substrate (*M*) which represents key anabolic driver species such as nucleotides, amino acids and ATP/GTP. tRNAs (*t*_*u*_) are produced and charged to *t*_c_ by a reaction which consumes *M*. All genes, enzymes (*E*), other host proteins (*Q*) and circuit genes are expressed in a three-step process: each gene (*j*) is bound by an RNA polymerase to form a transcription complex (*k*_*j*_) which undergoes transcription (*T*_*x*_), at a rate proportional to energy, to produce an mRNA (*m*_*j*_). The mRNA is then bound by a ribosome to form translation complex (*c*_*j*_) which undergoes translation (*T*_L_), consuming charged tRNAs (*t*_c_) in the process, to produce a protein (*p*_*j*_). We simplify the model dynamics by assuming that *k* and *C* species are at quasi-steady state, denoted with a ∼ overbar. The model explicitly considers resource biosynthesis. The RNA polymerase is produced like other host proteins. Ribosomes are produced in a three-step process. First, ribosomal proteins are produced in the same manner as other host proteins. Second, rRNAs are produced by transcription utilizing RNA polymerase, energy and nucleotides in the same manner as other host RNAs are produced. The ribosomal proteins then bind the rRNA to form functional ribosomes which are capable of translation. All reactions are described in detail in the Methods. Not all reactions are shown here in order to simplify the schematic.

Our model, calibrated to previously acquired batch culture data (Figure S1), is in qualitative agreement with our observations of cellular economy competition across low and high growth rates in a chemostat (Figure 1C). Simulations of the system dynamics from the mid-exponential phase steady state to the chemostat set growth rate show that growth rate initially falls from the maximal value obtained from batch culture and then slowly increases to reach the growth rate needed to match the chemostat dilution rate (Figure S2). The growth rate follows the proportion of the cellular resources (RNA polymerase and ribosomes) which slowly fall from their mid exponential phase peak to those seen in the chemostat.

Low dilution rates are often believed to be close to the stationary growth phase of a batch culture, which is known to trigger a stress response via the RpoS (σ^s^) regulon.^35^ Therefore, to assess whether low growth rates could play a role in the allocation of resources for gene expression, we monitored cellular stress linked to stationary phase using a plasmid encoded fusion between the stress responsive promoter *PbolA* and GFP.^36^ Our results indicated similar stress responses for all dilution rates tested (Figure 1D). This suggests that the macromolecular composition of the cell stemming from different dilution rates is largely independent of stress levels.

To investigate resource-mediated gene coupling, we utilized an E. coli strain that carries a plasmid encoding a constitutively expressed GFP and an inducible RFP under the control of LuxR (activated by acyl-homoserine lactone; AHL). This circuit enables a quantitative assessment of gene coupling and resource utilization as described previously.^8,12^ Briefly, in the absence of the inducer AHL, only the GFP reporter is produced. However, once AHL is added, this triggers the expression of RFP which competes with GFP for the same gene expression machinery. The two genes become coupled in the sense that as RFP increases, GFP decreases, and the production of each of them is constrained by a linear manifold.^8^ We analyzed gene couplings in the chemostat at the same growth rates as before using a range of AHL concentrations in each of those conditions. After five generations of growth, resulting in a physiologically steady state of the culture,^37^ the production of the reporters at each given growth rate was measured using flow cytometry.

When the chemostat was set at a 0.1 h^–1^ dilution rate, cells in the culture displayed a higher synthetic gene expression activity, compared to those in cultures with higher growth rates (Figure 3A). The expression levels of GFP and RFP were three- and four-fold higher, respectively, in cells with a 0.1 h^–1^ growth rate, compared to those in the culture set at a growth rate of 0.5 h^–1^. Cells grown in glucose-limited batch culture showed the highest growth rates but the lowest activity of the two reporters (Figure 3A). These results are in agreement with previous observations in batch culture^25^ and are supported by our resource allocation model (Figure 3B). We measured ribosome production by labeling the ribosomal L9 subunit with monomeric superfolder GFP (msfGFP) and found increased fluorescence with increased growth rate, reflecting a higher ribosomal protein synthesis (Figure 3C). The reduced synthesis of the reporters at high growth rates is not due to a lower production of ribosomes but rather due to higher ribosome production. Our model shows that as growth rate increases, cells invest more in the production of cellular resources, with RNA polymerase and ribosomes preferentially transcribing and translating their own components, and a concurrent decrease in circuit mRNA and protein synthesis (Figure 3D).

**Figure 3:**
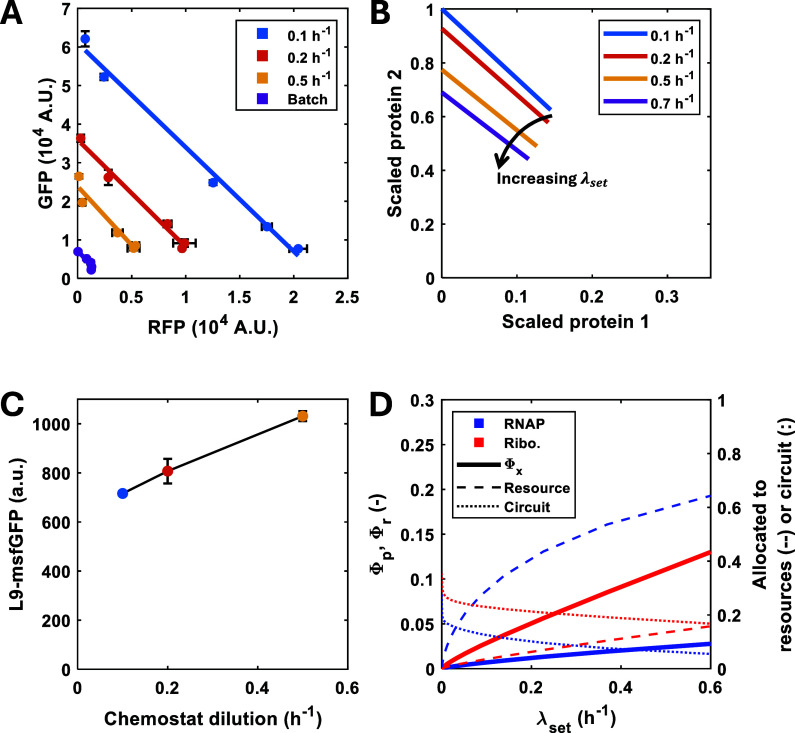
Growth-rate dependence of gene expression and resource allocation. (A) Resource mediated gene expression couplings were examined for different growth rates and AHL concentrations in cells carrying a genetic circuit that allows coexpression of GFP and RFP, which were determined by flow cytometry. Cells were also grown in batch cultures to quantify the reporter expression at the maximum growth rate. Points represent the mean and standard deviation (SD) of three biological replicates. (B) Simulations of the steady state concentration of the two gene circuit, normalized by maximum protein production, for different growth rates. Isocost lines were simulated by varying *u*_1_ between 0 and 1 while maintaining *u*_2_ = 1. Simulations were carried out as described in the Methods. (C) Ribosomal protein production was quantified by monitoring the expression, for different growth rates, of a genomic fusion of the ribosomal protein L9 to msfGFP. Bars represent the mean ± 1 SD *N* = 3. (D) Simulations of the cell’s internal resource economy expressing two circuit genes (with induction constants of *u*_1_ = 0, *u*_2_ = 1) over a range of growth rates. Φ_p_ and Φ*_r_* are the predicted mass factions of RNA polymerase and ribosomes, respectively.

We simulated synthetic circuit gene expression over a wide range of growth rates (Figure S3). At very low growth rates, protein production saturates as dilution rate falls to zero. This enables heterologous mass fraction to increase beyond experimentally observed values (Figure S3). While our model captures (p)­ppGpp regulation, in living cells increases in protein production of this magnitude are not observed due to physiological stress responses and other genetic changes which are not captured by our model (reviewed extensively elsewhere^38^).

Our experimental system also enabled us to test whether the growth rate affects gene expression in resource-mediated competition. As shown in Figure 3A, where the gene expression coupling occurred in continuous cultures, the expression level of GFP decreased when RFP accumulated in the cells regardless of the growth rate, and the concentration of the reporters is constrained by a linear manifold known as an “isocost line”.^8^ The same level of coupling, defined as the ratio of the trade-off in the expression between the two genes, was maintained in all the conditions tested (Figure 3A), suggesting that the distribution of costs of the protein production between transcription and translation (as determined by the slope of the linear manifold—a measure of the resources required per protein produced^8^) is independent of the growth rate and is governed only by the abundance of resources.

### Effect of Selective Inhibition of Transcription or Translation on Resource Allocation

Next, we sought to investigate the selective inhibition of components of the gene expression machinery to understand the effects of resource allocation at defined growth rates. To this end, sublethal concentrations of either rifampicin (*Rif*) or chloramphenicol (*Cm*) were added to the continuous culturing system for partial inhibition of RNA polymerases or ribosomes, respectively. Again, the use of chemostats allows us to untangle the impact of resource inhibition while forcing cells to maintain a fixed growth rate (which is not possible in a batch culture system).

When cells were treated with *Rif*, lower intensities of both GFP and RFP were recorded, with expression levels that gradually decreased with increasing concentrations of the antibiotic (Figure 4A). We modeled the inhibition of RNA polymerase by sequestering RNA polymerase through varying *k*_*rf*_ within the model (Figure 4B; see Supporting Information for details). As *k*_*rf*_ is increased, the number of RNA polymerases and ribosomes increases, as does the proportion of those dedicated to their own production (Figure 4D). This results in a concurrent fall in production of other genes; i.e., the proportion of RNA polymerases [ribosomes] transcribing [translating] circuit genes falls as the cell invests in resource biogenesis. This increase in resource production enables growth to be maintained at the set rate despite transcriptional inhibition (Figure 4C). We confirmed this prediction by treating cells grown at 0.1 h^–1^ carrying the L9 msfGFP fusion with *Rif*. This demonstrates increased synthesis of ribosomal proteins with increasing *Rif* addition (Figure 4C). Similarly, the production of both reporters decreased when a sublethal concentration of *Cm* was added to the culture (Figure S4), and our model predicts that as ribosomes are sequestered by an antibiotic, not only does the mass fraction of RNA polymerase and ribosome increase but so does the proportion of available resources which are utilized for resource production. Our model demonstrates that the feedback which is created by resource autocatalysis (i.e., that resources are required to make themselves) is sufficient to explain the fall in circuit expression which occurs during resource inhibition. We also found that partial inhibition of both transcription and translation at a fixed growth rate did not cause a significant change in coupling (i.e., the isocost gradients are constant), rather, there was only a change in the amount of circuit protein produced, indicating that reducing the transcription or translation activities does not change the cellular resource allocation regime.

**Figure 4:**
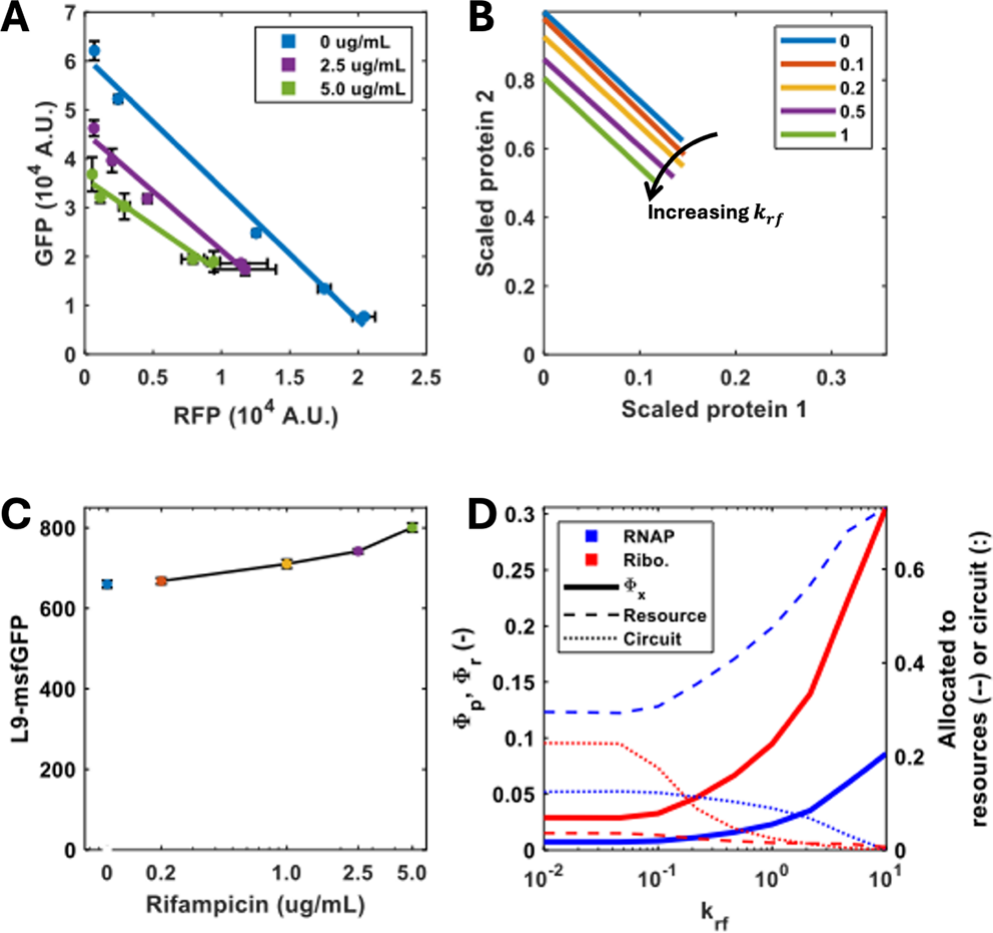
Effect of the selective inhibition of transcription on resource availability. (A) The strain carrying the GFP and RFP reporters was cultured in the minichemostat in the presence of sublethal concentrations of *Rif*. Points represent mean ± 1 SD *N* = 3. (B) Simulations of the steady state concentration of the two-reporter circuit normalized by maximum protein production, for different antibiotic transcriptional inhibition constants ranging from *k*_*rf*_ 0 to 0.1. Isocost lines were simulated by varying *u*_1_ between 0 and 1 while maintaining *u*_2_ = 1. Simulations were carried out as described in the Methods section. (C) The synthesis of the L9 ribosomal protein was examined in different concentrations of *Rif* at a fixed growth rate. Bars represent mean ± 1 SD *N* = 3. (D) Simulations of the cell’s internal resource economy expressing the two-reporter circuit (with induction constants of *u*_1_ = 0, *u*_2_ = 1) over a range of the RNA polymerase inhibition constant *k*_*rf*_ varied on a log10 scale from −2 to 1. Φ*_p_* and Φ*_r_* are the predicted mass factions of RNA polymerase and ribosomes, respectively.

### The Interplay Among rRNA Transcription, Growth, and Gene Expression

We investigated the effect of tuning the maximum size of the ribosomal pool on the protein production budget by using strains with successive deletions of the *rrn* operons.^39^ We transformed our resource-sensitive RFP-GFP circuit plasmid into an E. coli strain with four *rrn* deletions (SQ78) and found a decrease in gene expression as well as a reduction in the slope of the isocost lines (Figure 5A). Our model successfully captures these effects when the parameter *g*_*r*_ is tuned by the additional factor *D*_*r*_ (such that *g*_*r*_ = *D*_*r*_·*g*_*r*,0_) (Figure 5B).

**Figure 5:**
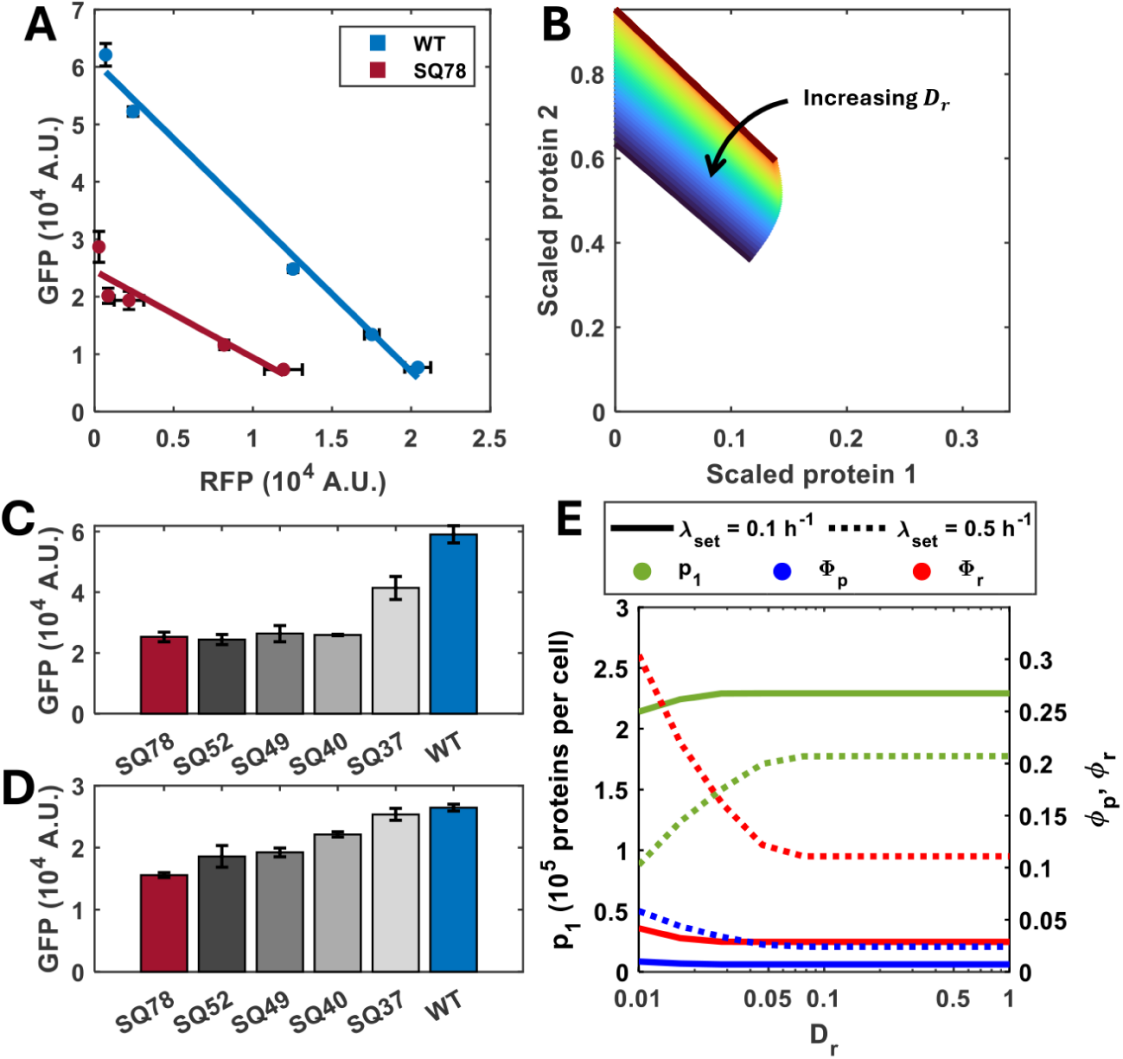
Translational capacity is dependent upon rRNA operon copy numbers. (A) The WT strain and SQ78 rRNA deletion strain carrying the GFP and RFP reporters were cultured in the minichemostat at a dilution rate of 0.1 h^–1^. Points represent mean ± 1 SD *N* = 3. (B) Simulations of the steady state concentration of the two-reporter circuit normalized by maximum protein production, for different *D*_*r*_ values. *D*_*r*_ tunes the rRNA copy number *g*_*r*_ as outlined in the methods. Isocost lines were simulated by varying *u*_1_ between 0 and 1 while maintaining *u*_2_ = 1. Simulations were carried out as described in the Methods section. (C) Constitutively expressed GFP levels were measured in strains carrying different numbers of *rrn* operons by growing them in the continuous culture low growth rate (0.1 h^–1^). The strains containing ribosomal deletions used in this experiment were (from left to right) SQ78, SQ52, SQ49, SQ40, SQ37 from ref 39 and the wildtype. Bars represent the mean ± 1 SD *N* = 3. (D) The same experiment as in (C) but carried out at a dilution rate of 0.5 h^–1^. (E) Simulations of the model showing the protein production (*p*_1_, green lines) and RNA polymerase (Φ*p*, blue lines) and ribosomal (Φ*_r_*, red lines) mass fraction for varied *D*_*r*_ values at two different *λ*_*set*_ rates (equivalent to dilution rates of 0.1 and 0.5 h^–1^, solid and dotted line styles respectively). Simulations were carried out as described in the Methods section. Φ*_p_* and Φ*_r_* are the predicted mass factions of RNA polymerase and ribosomes, respectively.

To further explore the relationship between rRNA copy number, gene expression and growth, we expressed the RFP-GFP circuit in multiple rRNA deletion strains cultivated in the chemostat at either 0.1 or 0.5 h^–1^ growth rates and used the constitutive GFP (i.e., AHL = 0 nM) as a reporter of translational capacity. When the production of GFP was analyzed, a higher accumulation of the protein was observed in the cells growing with the low dilution rate compared to the high dilution rate, regardless of their *rrn* copy number (Figure 5C compared to Figure 5D)— demonstrating again that growth and circuit gene expression are inversely correlated. The analysis also showed that the expression levels of GFP in fast-growing cells were weakly correlated with rRNA gene dosage (Figure 5D). In contrast, the synthesis of GFP was strongly dependent on the copy number of the *rrn* operon in slow-growing cells (Figure 5C). The synthesis of the fluorescent protein dropped by 30% and 60% in the strains containing one and two deleted *rrn* operons, respectively, compared to the wildtype strain growing at the same growth rate of 0.1 h^–1^. Nonetheless, only the two initial *rrn* deletions affected gene expression significantly, whereas the impact of further deletions was negligible (Figure 5C). Varying *D*_*r*_ in the model at low and high growth rates shows that this is due to the increased ribosome content of faster growing cells. Our model predicts that at low rRNA copy number, cells invest more resources in producing RNA polymerase and therefore can dedicate more transcriptional capacity to rRNA transcription (Figure 5E). This reduces the number of RNA polymerase available to transcribe circuit genes (Figure 5E).

### Metabolism and Translational Elongation Play a Key Role in the Allocation of Cellular Resources

Previous work has investigated nutrient limitations as one of the key factors controlling ribosome synthesis,^40,41^ although in these batch growth studies the contribution of nutrients is not isolated from the effect of changes in growth rate. To dissect the distinct contributions of growth rate and metabolism on gene expression, we grew the RFP-GFP reporter carrying strain at a set growth rate with different nutrient conditions. Samples were taken and circuit protein expression assessed by flow cytometry. At constant growth rate in glucose cultures, we found that increasing the nutrient richness by supplementing with casamino acids did not affect the expression of circuit genes (Figure S5). However, reducing nutrient quality by cultivating cells in glycerol, instead of glucose, led to a 30% reduction in the reporter activity, compared to that in the cells using glucose as the sole carbon source (Figure 6A). To identify the mechanistic cause of this fall in circuit expression we modeled the impact of changing the maximum tRNA production rate (*ψ*_*max*_) and the maximum peptide elongation rate (*γ*_*max*_). We found that varying both of these parameters alone is sufficient to recapitulate the experimental results, suggesting that the impact of changing carbon sources from glucose to glycerol reduces the availability of translational substrates (Figure 6B,D). Assessing the model’s internal cellular economy and elongation rates showed that as peptide elongation rate falls, the cell’s resources are redistributed (favoring ribosome production at the cost of circuit genes) in order to maintain growth rate. To maintain growth rate, the mass fraction of ribosomes rises as *D*_*γ*_ or *D*_*ψ*_ fall due to increased resource investment in resource production (Figure 6C,E). This increases the mass fraction of both RNA polymerase and ribosomes, reducing circuit gene expression. To corroborate this prediction, we measured the production of the L9 ribosomal protein and, as expected, glycerol-grown cells exhibited higher levels of fluorescence intensity relative to those in glucose-grown cells (Figure 6F). This result suggests that the lower expression of circuit genes observed in the cells grown in glycerol is due to a subtle interaction between elongation rate and number of ribosomes, and that growth rate does not set resource levels *per se*. Rather, growth rate sets the global translation rate, which is the product of the elongation rate and the number of translating ribosomes. As the elongation rate falls (e.g., due to the poorer quality substrate), the number of translating ribosomes must rise for growth to be maintained.

**Figure 6:**
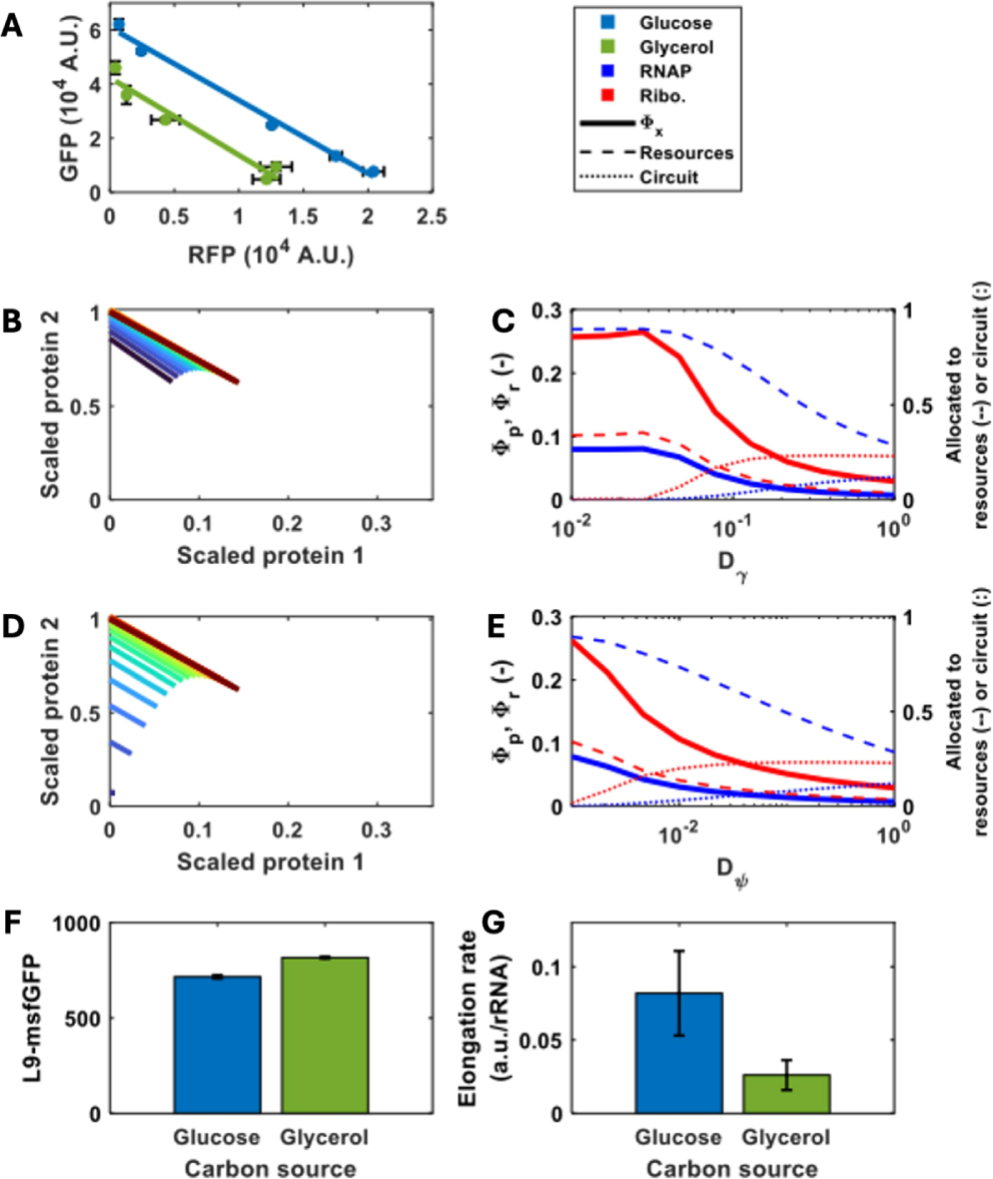
The effect of nutrient quality and peptide elongation on the allocation of cellular resources. (A) The expression capacity of the circuit genes was analyzed by comparing the data on the fluorescent intensity obtained from the dual fluorescent reporter strain grown either in glucose or in glycerol as the carbon source. (B) Simulations of the steady state concentration of the two-reporter circuit, normalized by maximum protein production, for different maximum peptide elongation rates. Change in peptide elongation rate are achieved by varying *D*_*γ*_ as detailed in the methods. Isocost lines were simulated by varying *u*_1_ between 0 and 1 while maintaining *u*_2_ = 1. Simulations were carried out as described in the Methods section. (C) Simulations of the steady state concentration of the two-reporter circuit, normalized by maximum protein production, for different maximum tRNA production rates. Changes in peptide elongation rate are achieved by varying *D*_*γ*_ as detailed in the methods. Isocost lines were simulated by varying *u*_1_ between 0 and 1 while maintaining *u*_2_ = 1. *Φ*_*p*_ and Φ_r_ are the predicted mass factions of RNA polymerase and ribosomes, respectively. Simulations were carried out as described in the Methods section. (D) Simulations of the cell’s internal resource economy and allocation of those resources for the two gene circuit (with induction constants of *u*_1_ = 0, *u*_2_ = 1) for various *D*_*γ*_ values. (E) Simulations of the cell’s internal resource economy and allocation of those resources for the two gene circuit (with induction constants of *u*_1_ = 0, *u*_2_ = 1) for various *D*_*ψ*_ values. Φ*_p_* and Φ*_r_* are the predicted mass factions of RNA polymerase and ribosomes, respectively. (F) The expression level of the L9 ribosomal protein fused to GFP was measured in the same culture condition as described in (A). Bars represent the mean ± 1 SD *N* = 3. (G) Elongation rate was measured in batch cultures as described in the Methods. Bars represent the mean slope of the elongation assay ± 1 SD *N* = 4. Means were calculated from means of technical replicates *N* = 3. * represents statistical significance as indicated by a Student’s *t* test with *p* = 0.01.

Our experimental results show that for the *same* growth rate, changing carbon source can manipulate the isocost lines, and our dynamic modeling results imply that this is due to a change in peptide elongation rate. This was confirmed experimentally, by conducting *in vitro* translation experiments using cell-free extracts obtained from cells that were grown in either glucose or glycerol as the sole carbon source. When these extracts were supplemented with the same amount of an mRNA coding for the fluorescent reporter mCherry, we identified a low but detectable signal that allowed to determine that translation rates (normalized by the amount of rRNA as a proxy for ribosome concentration) are twice as high in glucose compared to glycerol (Figures 6G and S6).

To investigate which underlying constraints within the E. coli metabolic network may impact translational precursors, we utilized flux balance analysis (details in the Methods) and flux variability analysis to identify metabolic nodes/subsystems which were most sensitive to the choice of carbon source. We augmented the iJO1366 reconstruction of the E. coli metabolic network^42^ with additional reactions to capture the amino acid and GTP consumption due to translation, using the amino acid sequence of GFP as a representative protein.^43^ For given glucose or glycerol exchange reaction [uptake] rates and different biomass production fluxes [growth rate], we optimized fluxes within the network to maximize GFP production. Given that FBA yields only a single solution and that often solutions to these optimization problems are poorly defined, we then carried out a flux variability analysis (FVA) to identify where fluxes may be “slack” i.e., able to take on a large range of possible values while maintaining the optimal protein output. This FBA and FVA modeling of the genome scale metabolic network showed that over a large range of biologically permissible carbon uptake rates and biomass production rates, glucose enables higher protein production (Figure 7A,B). We calculated the *net* rate through each subsystem in the metabolic network which shows that the models with glucose have greater flux through amino acid biosynthesis pathways (representative result shown in Figure 7C). Carrying out FVA and removing reactions from the analysis showed a large range of permissible values which suggests that most amino acid biosynthesis reactions have a narrow range of fluxes. Both carbon sources have a similar absolute flux through glycolysis/gluconeogenesis but glucose supports a high net flux through the pentose phosphate pathway. Our FVA analysis shows that while pyruvate metabolism can be more variable for the glycerol fed metabolic network than with a glucose input, the net flux through these pathways is over 100-fold higher (Figure 7C). We evaluated the total net flux though inflexible reactions which produce key metabolites for translation. Our analysis showed that the metabolic network with glucose input shows higher flux through amino acids and increased flux through ATP and NAD/NADH reactions (Figure 7D).

**Figure 7:**
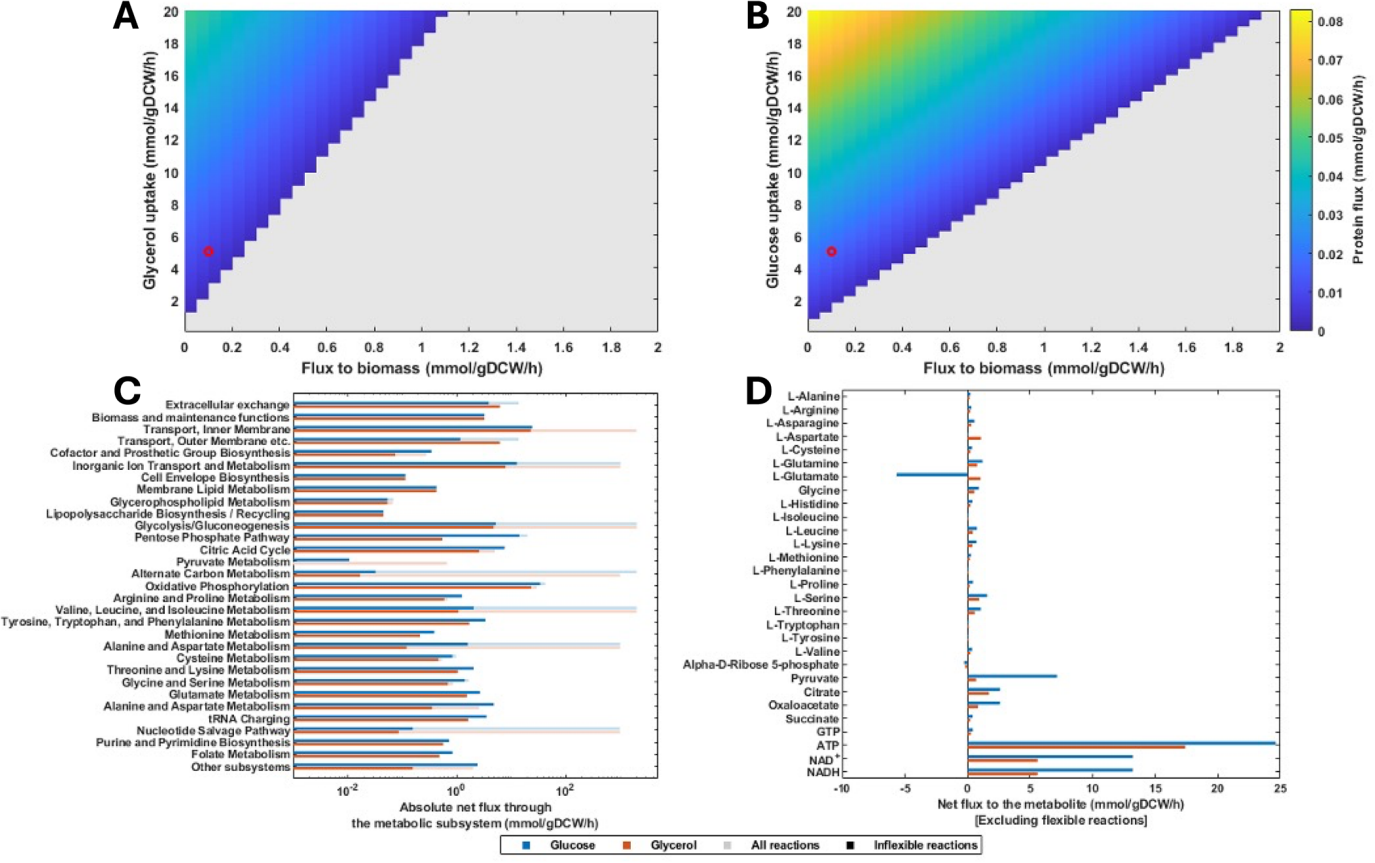
Genome scale metabolic modeling shows reduced protein production across a range of glycerol uptake rates and biomass production rates. A genome scale metabolic model was augmented with additional expressions describing the amino acid and GTP consumption of translation. The network was optimized for protein production and a flux variability analysis carried out as described in the Methods. (A) Maximal protein production flux produced by the metabolic network for the given glycerol uptake rate and biomass production rate. (B) Maximal protein production flux produced by the metabolic network for the given glucose uptake rate and protein rate. (C) The net flux through each subsystem of the metabolic network for the points highlighted in red in (A) and (B). The net flux was calculated as the sum (including positive and negative rates) for all the reactions in that subsystem. The flexible reactions were defined as those where absolute difference between the maximum and minimum fluxes produced by the FVA analysis was greater than 0.001 mmol/gDCW/h. The dark colored Inflexible Reactions bars are the sum of the rates through those reactions where the FVA predicted the maximum and minimum permissible fluxes were less than 0.001 mmol/gDCW/h. (D) The net flux through inflexible reactions producing and consuming the selected metabolites.

### (p)­ppGpp Affects the Global Distribution of the Proteome

Our findings show that the allocation of ribosomes to expendable proteins is inversely correlated with the growth rate, in agreement with the growth laws reported previously.^19–21,23,24^ A salient question is what are the effects of physiological mechanisms that are known to play a role in the allocation of cellular resources on this trade-off. Ribosome synthesis is mainly controlled by the alarmone, (p)­ppGpp,^18,20,21^ (p)­ppGpp accumulates at high levels when nutrients are limited, which results in the repression of both ribosomal proteins and rRNA production, and a decrease in growth rate,^18,20,21^ In E. coli, (p)­ppGpp is synthesized by RelA and degraded by the hydrolase SpoT.^17,21^ We studied the role of the alarmone on resource allocation for set growth rates, monitoring the activity of the RFP-GFP circuit in either *relA* or *spoT* deleted strains, exhibiting low or high (p)­ppGpp levels, respectively.^44,45^ When the mutant strains, carrying the reporter genes, were cultured in the chemostat at a growth rate of 0.1 h^–1^, a two-fold decrease in fluorescent intensity was recorded for both GFP and RFP in the *relA* deleted strain compared to its counterpart *spoT* mutant strain (Figure 8A).

**Figure 8:**
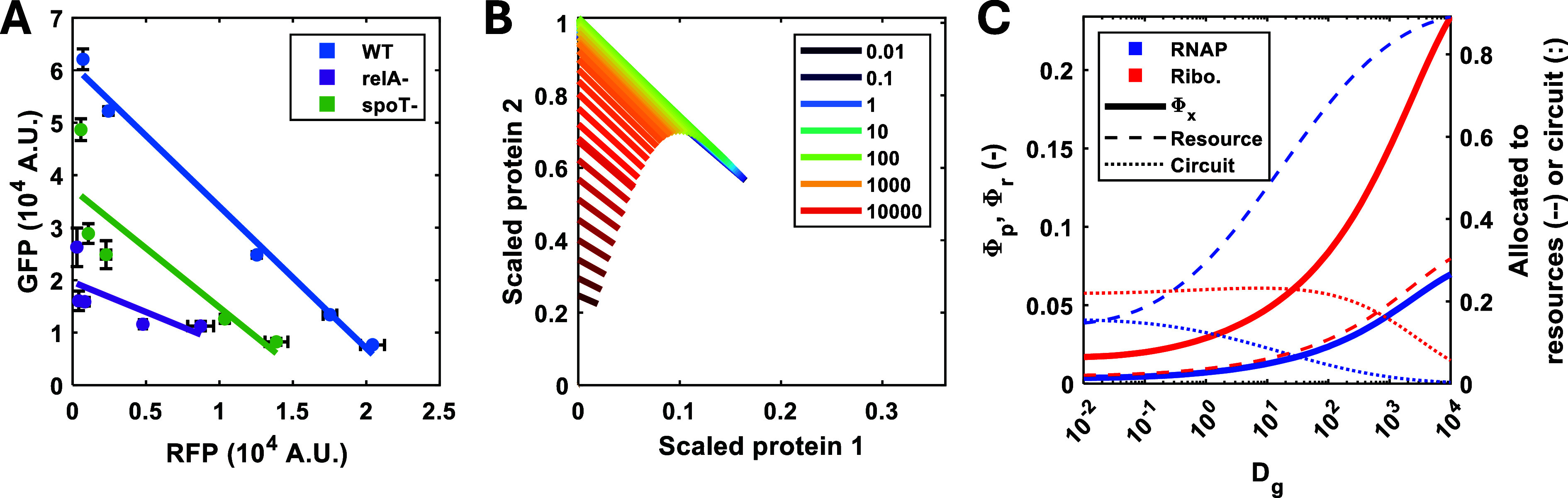
(p)­ppGpp mediated allocation of resources for gene expression. (A) Resource mediated gene expression coupling was examined in mutants of the (p)­ppGpp alarmone network. A *relA* mutant produces lower levels of (p)­ppGpp than the wildtype strain while the *spoT* mutant accumulates more (p)­ppGpp. Strains were cultured at a fixed growth rate in the minichemostat. Points represent the mean ± 1 SD *N* = 3. (B) Simulations of the steady state concentration of the two-reporter circuit, normalized by maximum protein production, for different (p)­ppGpp production rates. The impact of (p)­ppGpp regulation is modeled indirectly, with *D*_*g*_ tuning the strength of this regulation as detailed in the methods. Isocost lines were simulated by varying *u*_1_ between 0 and 1 while maintaining *u*_2_ = 1. Simulations were carried out as described in the Methods section. (C) Simulations of the cell’s internal resource economy and allocation of those resources for the two gene circuit (with induction constants of *u*_1_ = 0, *u*_2_ = 1) for various *D*_*G*_ values. Φ*_p_* and Φ*_r_* are the predicted mass factions of RNA polymerase and ribosomes, respectively.

This result suggests that the accumulation of the alarmone due to the mutation in the degradation activity encoded by *spoT* results in more resources being available for the circuit genes, due to a lower investment in ribosome synthesis. Interestingly, the RelA deficient strain showed a reduced resource-mediated coupling, which is associated with changes in the ratio between mRNAs and ribosome abundance (Figure 8A).^8^ This indicates that the alarmone is not only involved in controlling ribosome concentration, but also in global transcriptional suppression^20,46^ and thus the mutant strain exhibits a different cost of protein synthesis.^45^ To vary (p)­ppGpp in the model, we introduce a new parameter *D*_*g*_ which scales the (p)­ppGpp regulation variable. Increasing *D*_*g*_ is equivalent to decreasing (p)­ppGpp (modeling the proposed impact of the *relA* mutant) and vice versa (modeling the proposed impact of the *spoT* mutant). As *D*_*g*_ is varied we found that both the y-intercept and gradient of the isocost line is varied; increasing *D*_*g*_ (reducing (p)­ppGpp) decreases circuit gene expression (Figure 8B). This is due to a concurrent increase in resource production and a large rise in the proportion of resources dedicated to resource production (Figure 8C). Decreasing *D*_*g*_ (increasing (p)­ppGpp) from the nominal value (*D*_*g*_ = 1), is predicted to have a smaller impact on both the isocost line and the cell’s internal economy. Decreasing *D*_*g*_ reduces cellular production and increases the number of resources allocated to circuit gene expression (Figure 8C). However, this is not seen in our isocost line experiments, with the *spoT* deletion mutant suggesting that there may be additional impacts on gene regulation due to the removal of the SpoT protein, which is known to be essential in E. coli.^47^ The presence of compensatory mutations to mitigate this effect are not captured by a resource allocation model alone.

### Trade-Offs Between Growth and Metabolism Can Be Used to Optimize Bioprocesses

Our findings point to two key dials that can be easily tuned when designing bioprocesses. On one hand, we have demonstrated that the growth rate in the chemostat is responsible for setting up the budget of the cell by controlling the number of ribosomes available to engage with different processes. On the other hand, using a high-quality carbon source has the impact of increasing translation elongation rates. Hence, it logically follows that the combination of high ribosomes and high elongation rates should maximize bioproduction of a molecule of interest. We augmented the model with a metabolic pathway which utilizes the translational precursor *M* as a source and simulated the effects of changing chemostat dilution rate (Figure 9A). As growth rate increases, cellular resources (mass fraction of RNA polymerases and ribosomes) increase, which causes a concurrent decrease in enzyme production (Φ*_pathway_*). This is predicted to result in a decrease in metabolite production with increasing growth rate (Figure 8A). At very low rates our model predicts that enzyme production saturates and metabolite production reaches a peak and beings to fall (Figure S7). We experimentally verified the predicted growth/production relationship by conducting an experiment using glucose as a rich sole carbon source and comparing the production values of a strain carrying a plasmid for the biosynthesis of the antimicrobial and antitumoral drug violacein^12^ when cultured at different growth rates in the chemostat. Our results show that, as expected, slow growth renders higher production of total violacein in the reactor as well as a higher production per cell (Figure 9B).

**Figure 9:**
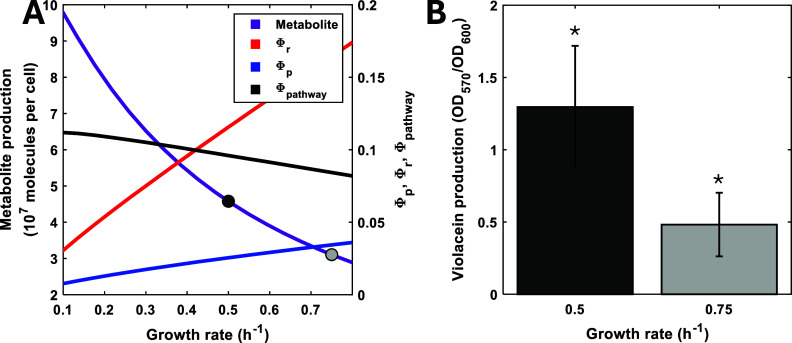
Violacein production is higher at lower growth rates. (A) The model was augmented with a metabolite pathway which drained the amino acid metabolite to a product representing violacein and simulated to steady state. Growth rate was varied as described in the Methods. The mass fraction of the pathway enzymes (Φ_pa*t*hway_), RNA polymerase (Φ_p_) and ribosomes (Φ_r_) are shown. The experimentally validated growth rates are highlighted in gray. Simulations were carried out as described in the Methods section. (B) Violacein per cell was measured by absorbance at 570 nm and normalized by cell density at 600 nm. Strains were cultured at a fixed growth rate in the minichemostat. Bars represent the mean ± 1 SD *N* = 3. * indicates statistical significance with *p* = 0.0415 in a two-sided Student’s *t* test.

## Discussion

In this study, we investigated how cellular resources are allocated to circuit gene expression under different cellular economic regimes. In artificial systems where ribosomal synthesis is not coupled to growth—e.g., cell-free extracts or cells endowed with orthogonal ribosomes—translational capacity for circuit genes is directly proportional to the size of the ribosomal pool. However, in natural systems, bacterial ribosomes preferentially synthesize more ribosomes to obtain rapid growth. This is at the expense of the production of other proteins, including those of synthetic circuits or pathways. This strategy is defined by bacterial growth laws which address the partitioning of the cellular proteome, as first proposed by Hwa and colleagues.^22–24^ In their original work, they cultivated E. coli cells in media containing varied carbon sources, resulting in different growth rates to obtain insights into the relation between the growth rate and the proteome distribution.

In this work we analyzed the specific contributions of metabolism and growth to the allocation of resources for gene expression. To this end, we exploited a continuous culture system that allowed us to manipulate the growth rate while maintaining the carbon source, to enable the investigation of *both* growth-dependent and growth-independent effects on resource allocation. Under growth-limited (i.e., growth controlled) conditions, we found lower expression levels of circuit genes in fast-growing cells relative to those in slow-growing cells, which is in line with previous observations from carbon-limited conditions. Our modeling analysis showed that this is the result of a greater allocation of cellular resources to the synthesis of gene expression machinery, which is required for cells to achieve maximum growth. This strategy enables cells to maximize reproductive fitness. It is noteworthy that the cost of protein synthesis (i.e., the resources required per protein produced) was essentially unchanged when cells redistributed resources, and thereby the level of resource mediated gene expression coupling was maintained.

This resource allocation strategy allows microbial cells to maintain cellular fitness in harsh conditions. For example, when transcription was partially inhibited with *Rif*, ribosomal protein synthesis increased, while the expression level of the circuit genes decreased. The model showed that this is the result of the self-investment of the gene expression machinery in its own synthesis to support the set growth rate. Similarly, we observed lower synthesis of the reporters in the presence of chloramphenicol-mediated translational inhibition. It is worth noting that although phenomenologically the cells behave in the same way, the specific regulatory mechanisms of resource (re)­allocation may vary depending on the point of inhibition. When treated with *Rf*, cells produce more ribosomes at the expense of the synthesis of other cellular RNAs, including circuit mRNAs, which leads to a reduced allocation of the proteome to heterologous genes. On the other hand, the inhibition of translation results in a reduced number of active ribosomes, as evidenced in previous reports conducting polysome profiling.^27,48^ Those studies show that when the cells are cultured in the presence of *Cm*, they accumulate 70S monosomes, which are not significantly involved in protein synthesis.

Our results show that this relationship between growth rate and resource allocation is also affected by ribosome biogenesis. We found that as the *rrn* operon copy number increased, circuit gene expression increases in both slow and fast-growing cells, but this effect is more pronounced in slow growth (where cells are investing less in resource production). The alarmone (p)­ppGpp also plays an important role by setting the number of resources available for the synthesis of circuit proteins. We determined that, when cultured at the same growth rate, a *relA* deficient strain, containing low (p)­ppGpp levels synthesized fewer heterologous proteins compared to a *spoT* mutant. Impairing RelA activity leads to reduced (p)­ppGpp production, potentially raising ribosome synthesis while impairing SpoT, leads to (p)­ppGpp accumulation and inhibition of ribosome synthesis.^17,18,21^ As ribosome synthesis falls, circuit gene expression rises, thus explaining the different behavior of these mutants.

We dissected the different contributions of growth rate and carbon source to the allocation of the cellular budget for gene expression. We determined that gene expression is not exclusively tuned by the number of ribosomes, but also depends on the peptide elongation rate. We found that increasing the quality of an already rich medium through amino acid supplementation did not change the translational capacity of the cell to produce the reporter proteins. On the other hand, when the cells metabolized glycerol, a poor-quality substrate, a lower expression of the circuit genes was observed compared to that in glucose (a high-quality carbon source *for* E. coli). These results are not due to a reduced availability of ribosomes in glycerol, and on the contrary, we found ribosomal protein synthesis was increased in glycerol. Instead, both our dynamic and genome scale metabolic modeling suggested that this is the result of a decrease in the translation elongation rates in the poor nutrient condition, a prediction which we then validated experimentally. In poor quality nutrients, such as glycerol, cells produce fewer translational building blocks—such as tRNA, elongation factor Tu, and guanosine triphosphate (GTP)^27^—and our flux balance analysis modeling suggests that poor quality nutrients result in an inefficient metabolism where carbon is sequestered away from amino acid biosynthesis and has poorer NADH and ATP yields. Our dynamic model predicts that to maintain an externally set growth rate in the presence of a lower elongation rate, cells must synthesize more ribosomes in order to maintain their total translational capacity. This results in a reduction in spare capacity and leading to a reduction in the synthesis of nonribosomal proteins.

We have formalized our above observations in a new host-circuit interaction model which captures the interplay between metabolism, transcription and translation of host and circuit genes, cellular resource biogenesis, (p)­ppGpp evolution and cell growth. While our model qualitatively captures most of our experimental observations, its definition of cellular metabolism remains simplistic with a focus on translational precursors making it only suitable for certain applications, such as violacein production. While the model captures heterologous protein burden (i.e., the fall in growth rate) its lack of explicit consideration of cellular responses (bar (p)­ppGpp regulation) means that protein production rates can be overestimated. Expanding the framework’s metabolism and stress response modeling is an area of ongoing work. In this work, we did not fit our model to our data set, instead fitting it to previously published data, and so our model does not show quantitative agreement with the experiments enclosed. Creating models with such prediction accuracy requires extensive and time-consuming characterization of host physiology which was out of scope for this study but is an area of ongoing research by ourselves and others (e.g. ref 49). Our framework makes also the usual assumptions of spatial homogeneity within the cell and lack of noise although new evidence suggests heterogeneity within the cytoplasm^50^ and noise have subtle but important impacts on interactions within gene circuits.^51^

The impact of host circuit interactions due to finite cellular resources has been identified as a cause of the failure of synthetic gene circuits since the field’s inception. Recently these interactions (notably in terms of ribosome-mediated coupling between coexpressed genes) have begun to be rigorously quantified. However, our results show that in addition to ribosomal competition, ribosome biosynthesis, the cell’s metabolism (mainly through ATP production and amino acid synthesis) and (p)­ppGpp are key drivers in setting the cell’s synthetic budget. These results have significant implications for the design of robust synthetic circuits and biotechnological pathways. For example, they provide a biological basis for strategies aimed at generating leaner proteomes^52^ or to decouple growth from production.^53^ Our results demonstrate that recombinant protein production can be increased by counterintuitive, yet simple, strategies such as artificially reducing growth rates and ensuring selection of carbon sources which facilitate maximal peptide elongation rates. We demonstrated the practical application of these findings by increasing the production of violacein three-fold using glucose as a substrate, simply by decreasing the dilution rate in the chemostat. It is worth noting that other factors need to be taken into consideration when designing the ideal bioprocess. For example, recent technoeconomic analyses show that crude glycerol can be a more cost-effective substrate compared to glucose syrup depending on the process, which may outweigh lower production titers.^54^ Moreover, the productivity of bioprocesses could be affected by reducing growth rates in chemostats, as it would take longer to reach the optimal biomass, although this could be mitigated by alternating between high and low dilution rates similarly to fed-batch processes.^55^ The conceptual framework and results described here represent a step toward a complete understanding of host-circuit interactions by including hitherto neglected aspects of cell physiology.

## Methods

### Minichemostat Setup

The custom-made mini continuous-culture system consisted of 15 reactors and was set following the protocol^37^ with some modifications. We used 80 mL glass bottles (VWR, Radnor, PA, USA) for the 50 mL cultures. We used silicone tubing (ID, 4 mm; OD 6 mm), 90 deg plastic tubes (ID, 4 mm; OD 6 mm), Norprene pump tubes (ID, 1.6 mm; OD, 5 mm, Sigma-Aldrich, St. Louis, MO, USA), plastic connectors, luer fittings, and syringe needles (14G and 19G) for the inflow and outflow of liquid cultures through a multichannel peristaltic pump (Cole-Parmer, St. Neots, UK). A magnetic bar was used in each reactor for continuous culture agitation with a microplate stirrer (2mag, Munich, Germany). The pump flow rate was set to 80, 230, and 400 μL·ml^–1^ to generate growth rates of, respectively, 0.1, 0.3, and 0.5 h^–1^.

### Cultivation of Bacterial Cells

Experiments in this study were conducted with E. coli MG1655 ^56^ and derivatives (Table S1). Strains were grown in batch or continuous cultures using M9 minimal medium (6 g·l^–1^ Na_2_HPO_4_, 3 g·l^–1^ KH_2_PO_4_, 1.4 g·l^–1^ (NH_4_)_2_SO_4_, 0.5 g·l^–1^ NaCl, 0.2 g·l^–1^ MgSO_4_·7H_2_O) supplemented with glucose (0.2%) and casamino acids (0.2%) at 30 °C, unless otherwise indicated. The antibiotics kanamycin (50 μg·ml^–1^), ampicillin (150 μg·ml^–1^) and gentamicin (20 μg·ml^–1^) were added when necessary.

Chemostat cultures carrying the pSEVA63-Dual plasmid were initiated from overnight cultures in batch. Cultures were induced from the beginning with N-acyl homoserine lactone (AHL, Sigma-Aldrich, St. Louis, USA, final concentrations of 0.5, 1, 5, 10 nM) and/or sublethal concentration of antibiotics such as *Rif* and *Cm* were added. After five generations of growth, when the population of cells in the chemostat reached a steady state, samples were collected to determine fluorescent reporter expression levels (see below). E. coli MG1655 wild-type as well as the strains carrying the L9-*msf*GFP harboring the L9 ribosomal protein labeled with GFP and the *PbolA-GFP* fusion used to determine intracellular stress, were cultured in the minichemostat without inducers.

### Determination of Intracellular Components

Samples of 0.5–2 mL of culture were collected from the chemostat at the steady state. Cells were pelleted by centrifugation and 1 mL of RNAProtect Bacteria Reagent (Qiagen, Venio, Netherlands) was added prior to freezing in ethanol and dry ice and then stored at −80 °C. RNA was purified with a RNeasy Kit (Qiagen, Venio, Netherlands) following the protocol of the manufacturer. An additional on-column DNA digestion was performed during the extraction with RNase-Free DNase Set (Qiagen, Venio, Netherlands). DNA was purified using a Monarch Genomic DNA Purification Kit (New England Biolabs, Ipswich, USA), according to the instructions of the manufacturer. Total RNA and DNA concentrations were quantified with a Tecan Infinite M200 Microplate Pro Reader (Tecan, Männerdorf, Switzerland). rRNA was determined using a TapeStation system (Agilent, Santa Clara, USA) by loading 1 μL of RNA into an Agilent RNA screen tape following the instructions of the manufacturer. Proteins were isolated by resuspending the sample frozen pellets in 100 μL of B-PERTM Bacterial Proteins Extraction Reagent (Thermo Fisher, Waltham, USA) and allowing the reagent to act for 15 min. Cell debris was then removed by centrifugation for 10 min at 10,000 rpm and 5 μL of supernatant were used in a microplate Quick StartTM Bradford Protein Assay (Bio-Rad, Hercules, USA). Protein concentration was determined by reading the absorbance at 559 nm of preparations in a flat 96-well plate with Tecan Infinite M200 Microplate Pro Reader (Tecan, Männerdorf, Switzerland) and interpolating the values in a standard curve of known concentrations of bovine serum albumin (BSA). Experimental values shown in the figures correspond the mean of three independent biological replicates obtained from three technical replicates each.

### Cloning Procedures and Construction of Reporter Strains

The characteristics of the bacteria, plasmids and primers used in this study are described in Supplementary File 1 Table S1. DNA manipulation was carried out following standard protocol.^57^ Plasmid DNA was isolated from bacterial cells using a commercial QIAprep Spin Miniprep Kit (Qiagen, Venio, Netherlands). Restriction endonucleases were purchased from New England Biolabs (NEB, Ipswich, MA, USA).

The E. coli MG1655-derived strains containing the *rplI­(L9)-msfGFP* fusion was constructed with a seamless allelic replacement method described previously.^58^ The delivery plasmid pEMG was used for the genomic exchange *rplI* x *rplI*-*msf*GFP and was built as follows: First, the entire *rplI* gene (TS1*^rplI^*, ∼0.5 kb) and downstream (TS2*^rplI^*, ∼0.5 kb) regions of the 3′-end of the gene were amplified with primer pairs rplI-TS1F/R and rplI-TS2F/R respectively (Table S1). Another DNA segment bearing the *msfGFP* sequence flanked by overlapping regions with those of the TS1*^rplI^* and TS2*^rplI^* fragments was prepared by amplification of plasmid pBG with primers rplI-msfGFP-F/R. The resulting TS1*^rplI^*, *msfGFP* and TS2*^rplI^* were then joined by isothermal assembly^59^ and cloned in pEMG (Table S1) previously digested with *Eco*RI and *Bam*HI, to yield pEMG-*rplI*-*msfGFP*, transformed into E. coli DH5α λ *pir*. This plasmid was transferred to E. coli MG1655 where it can not replicate and was cointegrated in the chromosome. Next, the pSW plasmid that expresses I-SceI endonuclease under the *Pm* promoter^43^ was introduced by electroporation into the pEMG-*rplI*-*msf*GFP cointegrated. Selected clones were grown in LB medium with Ap and 3MBz (5 mM) to activate the *Pm* promoter, allowing I-SceI expression, which in turns forces a second recombination event by cleaving the cointegrate. Cells were plated on LB agar and the generation of a chromosomal *rplI*-*msf GFP* fusion was confirmed by testing the loss of the pEMG-*rplI*-*msf* GFP-encoded Km resistance gene. Km-sensitive clones were selected and chromosomal replacement further confirmed by PCR with oligonucleotides rplI-TS1F/TS2R (Supplementary Table S1). pSW was finally cured by serial dilutions in the absence of antibiotics, yielding the strain E. coli MG1655 *rplI-msf GFP.*

To inactivate the SpoT activity in the BW25113 strain, the target genomic region was replaced with a kanamycin antibiotic cassette (pKD4) using primers spoT KO F/-R, following a method described previously^60,61^ and the genomic deletions were further confirmed by PCR.

### Fluorescence Measurements

Intracellular abundance of fluorescence proteins was determined with an Attune NxT Flow Cytometer (ThermoFisher, Waltham, USA) analyzing GFP and RFP expression using blue (excitation 488 nm; emission 530/30 nm) and yellow (excitation 561 nm; emission 620/15 nm) lasers, respectively. 100 μL of the sample was taken from each culture and mixed with 200 μL of PBS prior to injecting into the flow cytometer. 30,000 events of each sample were analyzed to determine population means using the default software of the instrument. Experimental values shown in the figures were determined by calculating the mean of three independent biological replicates conducted on different days (cultures grown from different colonies), each obtained from the mean of three technical replicates (cultures grown from the same colony).

### Determination of Translation Rates

E. coli MG1655 lysates were prepared by adapting the method described in ref 62. Cells were grown at 37 °C with 250 rpm shaking and up to an optical density at 600 nm of 2–2.5 in 250 mL of M9 supplemented with the respective carbon source (glucose or glycerol). Cell pellets were recovered by centrifugation for 5 min at 12,000 g and lysed using a sonicator (Fisher Scientific Model FB120) at 20 MHz with an amplitude of 50% in 10 cycles of 10 s separated by 1 min incubation on ice until reaching a total energy of 440–480 J. The resulting lysate was cleared by centrifugation at 4 °C and 13,400 rpm for 10 min, followed by a runoff step of 20 min at 37 °C with 250 rpm shaking to preactivate the lysate. This was followed by another centrifugation step (same conditions) before freezing at −80 °C until use.

The translation reactions were conducted by adding 50 nM of mCherry mRNA in a maximum volume of 2 μL and supplementing the extracts with magnesium glutamate 8 mM, potassium glutamate 180 mM and PEG8000 2% for a total reaction volume of 10.5 μL. Amino acid or energy mixes were omitted in all samples except in the positive control of E. coli BL21 grown in LB. Reactions were set up in a 384 well plate (Greiner Bio-One small volume) and kinetics were tracked in a CLARIOstarPlus plate reader (BMG Labtech) for 12 or 24 h at 37 °C reading the mCherry signal every 5 min (exc. 570 nm/em. 620 nm; gain 3,000). The translation rate was calculated by fitting the reaction curve using linear regression. Reported rates correspond to the mean of 4 biological replicates with 3 technical replicates each.

### Violacein Production

Violacein production was conducted as reported previously.^12^ E. coli MG1655 cells were grown in M9 minimal medium supplemented with glucose at different dilution rates for 24 h. Cells present in 1 mL of culture were recovered by centrifugation (13,000 rpm, 10 min). Violacein was extracted from the cell pellets with absolute ethanol incubating at 95 °C for 10 min and it was determined reading absorbance at 570 nm. Values were normalized by the amount of biomass present in the cell debris after extraction determined spectrophotometrically at 600 nm.

### Derivation of the Mathematical Model

The model couples mechanistic models of transcription and translation with phenomenological descriptions of microbial growth and a simple representation of metabolism to produce a tractable model capable of recapitulating the different distributions of cellular proteins (such as transporters, enzymes, host factors, RNA polymerase, ribosomes and circuit genes) in addition to partitions of RNAP for transcription of r or mRNA coding genes. Applying the Law of Mass Action to the reactions listed below creates a single host model composed of coupled ordinary differential equations capturing the following universal constraints:

i Finite biosynthetic capacity provided by a simple metabolism
  - Metabolic enzymes create limitation in nutrient uptake and production of anabolic drivers (e.g., amino acids, nucleotides, ATP)
  - Protein production consumes these anabolic drivers
  - External nutrients can also be constrained allowing modeling of (i) midexponential growth, (ii) batch culture and (iii) chemostat
ii Total proteome mass is constant
iii Competition for RNA polymerases by mRNA and rRNA promoters, including the impact of increasing copy number with growth rate
iv Competition for ribosomes by different mRNAs
v Resource biosynthesis
  - Including autocatalysis—i.e., gene expression resources are required for their own production
  - Including the underlying metabolic limitations on reaction rates
vi Dynamic feedback between growth and resource biosynthesis

We consider growth in the presence of an external substrate *S* which is imported into the cell and converted into the intermediate small molecules (denoted *M*) by enzymes. The external substrate enters the system at rate kin and is removed at rate δ. Considering metabolism and growth in this way allows modeling of:

i Single cells during exponential growth (where the external resource is constant and population dynamics are ignored, i.e., *dN*/*dt* and *dS*/*dt* are set to zero)
ii Batch culture (where the external resources deplete and the population grows over time, where *k*_*in*_ = δ = 0)
iii Growth in a chemostat (where at steady state d*N*/d*t* = 0 when the growth rate λ equals the dilution rate δ)

This allows the model to replicate the multiple culture methods available. The dynamics of the external substrate are

$$\frac{dS}{dt}=\{\begin{array}{cc} \underset{\mathrm{constant external substrate}}{\underset{︸}{0}} & \mathrm{Single cell dynamics only mode}\\ \underset{\mathrm{consumption of}s\mathrm{by}N\;\mathrm{bacteria}}{\underset{︸}{-\left(\frac{v_{E}\cdot S\cdot p_{E}}{\kappa_{E}+S}\right)\cdot N}} & \mathrm{Batch culture model}\\ \underset{s\mathrm{influx}}{\underset{︸}{k_{in}\;}}-\underset{\mathrm{consumption of s by}N\;\mathrm{bacteria}}{\underset{︸}{\left(\frac{v_{E}\cdot S\cdot p_{E}}{\kappa_{E}+S}\right)\cdot N}}-\underset{\mathrm{s efflux}}{\underset{︸}{\delta \cdot S}} & \mathrm{Chemostat model} \end{array}$$

The dynamics of the population depend on the culture conditions and are given by

$$\frac{dN}{dt}=\{\begin{array}{ll} 0 & \mathrm{Single cell dynamics only model}\\ \lambda \cdot N & \mathrm{Batch culture model}\\ \left(\lambda -\delta \right)\cdot N & \mathrm{Chemostat model} \end{array}$$

The substrate *S* is converted into the intermediate small molecules substrate (such as amino acids, nucleotides and ATP), denoted *M*. Each s molecule yields *ϕ M* molecules. *M* molecules are consumed by charging tRNAs.

$$\frac{dM}{dt}=\underset{\mathrm{conversion}\;\mathrm{of}\;S\;\mathrm{to}\;M}{\phi \cdot \underset{︸}{\left(\frac{v_{E}\cdot S\cdot p_{E}}{\kappa_{E}+S}\right)}}-\underset{\mathrm{charging}\;\mathrm{of}{\;t}_{u}\;\mathrm{by}\;M}{\underset{︸}{\left(\frac{v_{T}\cdot p_{E}}{1+\frac{K_{u}}{t_{u}}+\frac{K_{M}}{M}}\right)}}-\underset{\mathrm{dilution by growth}}{\underset{︸}{\lambda \cdot M}}$$

Uncharged tRNAs *t*_*u*_ are born at a maximum rate *ψ*_*max*_, which is scaled by the ribosome biosynthesis regulation function *R*_*r*_, and are released by translation. Therefore, the dynamics of the uncharged tRNAs are

$$\frac{dt_{u}}{dt}=\underset{\begin{array}{c} spontaneous\;tRNA\;\\ production \end{array}}{\underset{︸}{\psi_{max}\cdot R_{r}}}-\underset{\mathrm{charging of}\;t_{u}\;\mathrm{by}\;M}{\underset{︸}{\left(\frac{v_{T}\cdot p_{E}}{1+\frac{K_{u}}{t_{u}}+\frac{K_{M}}{M}}\right)}}+\underset{\begin{array}{c} production\;of\;free\;tRNAs\;\\ by\;translation \end{array}}{\underset{︸}{\gamma_{X}\left(t_{c}\right)\cdot \sum_{j}\left(c_{j}\right)}}-\underset{\mathrm{dilution by growth}}{\underset{︸}{\lambda \cdot t_{u}}}$$

The uncharged tRNAs *t*_*u*_ are charged by reaction with *M* to produce charged tRNAs *t*_*c*_. This reaction captures the ATP and amino acid dependent nature of tRNA charging. Charged tRNA *t*_*c*_ are consumed by translation and dilute due to cell growth. The full dynamics of *t*_*c*_ are

$$\frac{dt_{c}}{dt}=\underset{\mathrm{charging of}\;t_{u}\;\mathrm{by}\;M}{\underset{︸}{\left(\frac{v_{T}\cdot p_{E}}{1+\frac{K_{u}}{t_{u}}+\frac{K_{M}}{M}}\right)}}-\underset{\begin{array}{c} consumption\;of\;tRNAs\\ by\;translation \end{array}}{\underset{︸}{\gamma_{X}\left(t_{c}\right)\cdot \sum_{j}\left(c_{j}\right)}}-\underset{\mathrm{dilution by growth}}{\underset{︸}{\lambda \cdot t_{c}}}$$

Messenger RNAs *m*_*j*_ of protein-encoding genes are produced from transcription complexes ${\mathrm{k̃}}_{j}$ in transcription and are lost by decay and dilution:

$$\frac{dm_{j}}{dt}=\underset{\mathrm{transcription}}{\underset{︸}{\tau_{j}\left(M\right)\cdot {\mathrm{k̃}}_{j}\;}}-\underset{\mathrm{decay}}{\underset{︸}{\delta_{m}\cdot m_{j}\;}}-\underset{\mathrm{dilution by growth}}{\underset{︸}{\lambda \cdot m_{j}}}$$

Proteins *p*_*j*_ are produced from translation complexes ${\mathrm{c̃}}_{j}$ and lost due to dilution due growth:

$$\frac{dp_{j}}{dt}=\underset{\mathrm{translation}}{\underset{︸}{\gamma_{j}\left(M\right)\cdot {\mathrm{c̃}}_{j}\;}}-\underset{\mathrm{dilution by growth}}{\underset{︸}{\lambda \cdot p_{j}}}$$

The transcription complexes ${\mathrm{k̃}}_{j}$ and translation complexes ${\mathrm{c̃}}_{j}$ are composed of promoters bound by RNA polymerase and mRNAs bound by ribosomes, respectively. We assume that these reactions are at quasi-steady state (as others have done previously.^4,30^

The concentration of ${\mathrm{k̃}}_{j}$ :

$${\mathrm{k̃}}_{j}=\frac{K_{j,X}\cdot P_{tot}}{1+\sum \left(K_{i,X}\right)}$$

where *K*_*j,X*_ is the RNAP sequestration potential of gene *j* and *K*_*i,X*_ is the potential of all the RNAP consuming genes (*i* = {*E*, *Q*, *P*, *R*, *r*, 1, 2}). This expression is similar to those derived by ref 4 and in ref 11

$$K_{j,X}=\frac{g_{j}}{\kappa_{X,j}}\cdot R_{j}\left(\cdot \right)$$

where *g*_*j*_ is the effective copy number of the gene. We model the effective copy number as proportional to growth rate to account for pseudocopy number expansion at high growth rate. *g*_*j*_ is calculated algebraically as

$$g_{j}=g_{0}\cdot \exp\left(q_{x}\cdot \lambda \right)$$

where *g*_*0*_ is the copy number per chromosome and the exponential captures the additional copy number expansion as growth rate increases. *κ*_*X,j*_ is the promoter-RNA polymerase dissociation constant:

$$\kappa_{X,j}=\frac{k_{p,j,r}+\tau_{j}\left(M\right)+k_{rf}}{k_{p,j,f}}$$

where *k*_*p,j,r*_ is the unbinding rate of the RNA polymerase from the promoter, *τ*_*j*_ (*M*) is the nucleotide elongation rate, *k*_*rf*_ is the *Rif* sequestration rate and *k*_*p,j,p*_ is the RNAP-promoter binding rate. The $R_{j}\left(\cdot \right)$ function captures the regulation of the gene and is unique for each *j*, see further description below.

The concentration of ${\mathrm{c̃}}_{j}$ is

$${\mathrm{c̃}}_{j}=\frac{K_{j,L}\cdot R_{tot}}{1+\sum \left(K_{i,L}\right)}$$

where *K*_*j,L*_ is the ribosome sequestration potential of mRNA *j m*_*j*_ and *K*_*i,L*_ is the potential of all the ribosome utilizing genes (*i* = {*E*, *Q*, *P*, *R*, 1, 2}).

This expression is similar to those derived by,^4,11,30^

$$K_{j,L}=\frac{m_{j}}{\kappa_{L,j}}$$

where *κ*_*L,j*_ is the mRNA-ribosome dissociation constant:

$$\kappa_{L,j}=\frac{k_{r,j,r}+\gamma_{j}\left(t_{c}\right)+k_{cm}}{k_{r,j,f}}$$

And *k*_*r,j,r*_ is the unbinding rate of the ribosome from the mRNA, *γ*_*j*_ (*t*_*c*_) is the peptide elongation rate, *k*_*cm*_ is the *Cm* sequestration rate and *k*_*r,j,f*_ is the ribosome mRNA binding rate.

The dynamics of the total RNA polymerase *P*_*tot*_ follow the same dynamics as the enzymes and host proteins but with the added impact of sequestration by the transcriptional inhibitor.

$$\frac{dP_{tot}}{dt}=\underset{\begin{array}{l} RNAP\\ translation \end{array}}{\underset{︸}{\gamma_{p}\cdot c_{p}\;}}-\underset{\mathrm{dilution}\;\mathrm{by}\;\mathrm{growth}}{\underset{︸}{\lambda \cdot P_{tot}}}-\underset{\begin{array}{l} sequestration\\ \;by\;antibiotic \end{array}}{\underset{︸}{k_{rf}\cdot P_{tot}\;}}$$

The rRNA *r* is produced by transcription and diluted due to growth. The free rRNA binds (reversibly) to r-proteins to produce the total pool of functional ribosomes *R*_*tot*_.

$$\frac{dr}{dt}=\underset{\mathrm{transcription}}{\underset{︸}{\tau_{r}\left(M\right)\cdot k_{r}\;}}-\underset{\mathrm{dilution by growth}}{\underset{︸}{\lambda \cdot r}}-\underset{\begin{array}{l} \mathrm{ribosome}\\ \mathrm{assembly} \end{array}}{\underset{︸}{\beta_{\rho }\cdot p_{r}\cdot r\;}}+\underset{\begin{array}{l} \mathrm{ribosome}\\ \mathrm{disassembly} \end{array}}{\underset{︸}{\mu_{\rho }\cdot R_{tot}\;}}$$

The dynamics of the r-protein genes includes their mRNA *m*_*r*_ and protein *p*_*r*_. The *m*_*r*_ dynamics follow the same dynamics as other genes. The r-proteins are produced by translation from the *c*_*r*_ complex and bind to the rRNA *r* giving the following dynamics:

$$\frac{dp_{r}}{dt}=\underset{\begin{array}{l} \mathrm{r-}\mathrm{protein}\\ \mathrm{translation} \end{array}}{\underset{︸}{\gamma_{r}\cdot c_{r}\;}}-\underset{\mathrm{dilution by growth}}{\underset{︸}{\lambda \cdot p_{r}}}-\underset{\begin{array}{l} \mathrm{ribosome}\\ \mathrm{assembly} \end{array}}{\underset{︸}{\beta_{\rho }\cdot p_{r}\cdot r\;}}+\underset{\begin{array}{l} \mathrm{ribosome}\\ \mathrm{disassembly} \end{array}}{\underset{︸}{\mu_{\rho }\cdot R_{tot}\;}}$$

The dynamics of the total functional ribosome pool *R*_*tot*_ follow:

$$\frac{dR_{tot}}{dt}=\underset{\begin{array}{l} \mathrm{ribosome}\\ \mathrm{assembly} \end{array}}{\underset{︸}{\beta_{\rho }\cdot p_{r}\cdot r\;}}-\underset{\begin{array}{l} \mathrm{ribosome}\\ \mathrm{disassembly} \end{array}}{\underset{︸}{\mu_{\rho }\cdot R_{tot}\;}}-\underset{\mathrm{dilution}}{\underset{︸}{\lambda \cdot R_{tot}}}-\underset{\begin{array}{l} sequestration\\ \;by\;antibiotic \end{array}}{\underset{︸}{k_{cm}\cdot R_{tot}\;}}$$

The dynamics of the inhibited RNA polymerase *P*_*ab*_ and ribosome *R*_*ab*_ follow:

$$\frac{dP_{ab}}{dt}=\underset{\begin{array}{l} sequestration\\ \;by\;\mathrm{antibiotic} \end{array}}{\underset{︸}{k_{rf}\cdot P_{tot}\;}}-\underset{\mathrm{dilution by growth}}{\underset{︸}{\lambda \cdot P_{ab}}}$$

$$\frac{dR_{ab}}{dt}=\underset{\begin{array}{l} \mathrm{sequestration}\\ \;by\;\mathrm{antibiotic} \end{array}}{\underset{︸}{k_{cm}\cdot R_{tot}\;}}-\underset{\mathrm{dilution by growth}}{\underset{︸}{\lambda \cdot R_{ab}}}$$

The global mRNA elongation rate *τ*_*X*_ (*M*) is proportional to the number of metabolites *M* in the cell:

$$\tau_{X}\left(M\right)=\frac{\tau_{max}\cdot M}{K_{\tau }+M}$$

The elongation rate *τ*_*j*_ of a specific mRNA *m*_*j*_ is determined by its length (in amino acid codons *n*_*j*_):

$$\tau_{j}\left(M\right)=T_{X\left(M\right)}/\left(3\cdot n_{j}\right)$$

The elongation rate *τ*_*r*_ of the rRNA *r* is given by

$$\tau_{r}=\tau_{r,max}/n_{r}$$

where *τ*_*r,max*_ is the maximal elongation rate and *n*_*r*_ is the length of the rRNA in nucleotides.

The global peptide elongation rate γ­(*t*_*c*_) is proportional to the number of charged tRNAs in the cell:

$$\gamma_{X}\left(t_{c}\right)=\frac{\gamma_{max}\cdot t_{c}}{K_{\gamma }+t_{c}}$$

The elongation rate *γ*_*j*_ of a specific protein *p*_*j*_ is determined by its length (in amino acids *n*_*j*_):

$$\gamma_{j}\left(t_{c}\right)=\gamma_{X}\left(t_{c}\right)/n_{j}$$

We model gene regulation $R_{j}\left(\cdot \right)$ phenomenologically based on a species which approximates the regulation by (p)­ppGpp. Taking inspiration from,^30^ we define θ as the ratio between the charged and uncharged tRNAs:

$$\theta =t_{c}/t_{u}$$

This is effectively the inverse of the (p)­ppGpp concentration (see^30^ for full discussion).

Given that most E. coli are subjected to negative feedback in some way we model the regulation of the house keeping “q” genes using a negative Hill function

$$R_{Q}\left(p_{Q}\right)=\frac{1}{1+\left(\frac{p_{Q}}{\kappa_{q}}\right)}$$

­(p)­ppGpp accumulation inhibits cellular resource biosynthesis and therefore we model the regulation of the RNA polymerase, r-proteins and rRNA as follows:

$$R_{p}\left(\theta \right)=R_{r}\left(\theta \right)=R_{rr}\left(\theta \right)=\frac{\theta }{\theta +\kappa_{\theta }}$$

ppGpp accumulation also activates enzyme protein through interactions with the RNA polymerase:^21^

$$R_{E}\left(\theta \right)=\frac{\kappa_{\theta }}{\theta +\kappa_{\theta }}$$

We model cell growth rate λ as in^31^

$$\lambda \left(t_{c}\right)=\left(\frac{1}{M_{0}}\right)\cdot \gamma_{X}\left(t_{c}\right)\cdot \underset{j}{\sum }\left({\mathrm{c̃}}_{j}\right)$$

where *γ*_*X*_ (·) is the global peptide elongation rate, *M*_0_ is the midexponential phase cell mass and ∑*c*_*j*_ is the sum of the translating ribosomes.

### Incorporating Circuit Gene Expression and Protein Production

We introduce circuit genes by introducing new species and equations describing the production of transcription complexes, mRNA, translation complexes and proteins. These equations show the same dynamics as described above in “Derivation of the mathematical model”. The definition of growth rate λ is updated to include the additional translation complexes of the new genes. We model the two gene RFP-GFP resource competition reporter circuit by introducing two new genes. Each new gene *i* is regulated as follows:

$$R_{i}=\left(\frac{\kappa_{theta}}{\theta +\kappa_{\theta }}\right)\cdot u_{i}$$

where *u*_*i*_ takes a value between 0 and 1 and scales the RNAP-promoter association rate.

To introduce the metabolic pathway, we introduce a new metabolite *X* which is produced from the metabolite *M*. Therefore, in addition to incorporating the RNA polymerase and ribosome consumption, to account for the synthesis of the new enzymes *dM*/*dt* is modified to

$$\frac{dM}{dt}=\underset{\mathrm{conversion}\;\mathrm{of}\;s\;\mathrm{to}\;M}{\underset{︸}{\phi \cdot \left(\frac{v_{E}\cdot S\cdot p_{E}}{\kappa_{E}+S}\right)}}-\underset{\mathrm{conversion}\;\mathrm{of}\;M\;\mathrm{to}\;X}{\underset{︸}{\left(\frac{v_{ME}\cdot M\cdot p_{1}}{\kappa_{ME}+M}\right)}}-\underset{\mathrm{charging}\;\mathrm{of}{\;t}_{u}\;\mathrm{by}\;M}{\underset{︸}{\left(\frac{v_{T}\cdot p_{E}}{1+\frac{K_{u}}{t_{u}}+\frac{K_{M}}{M}}\right)}}-\underset{\mathrm{dilution by growth}}{\underset{︸}{\lambda \cdot M}}$$

where *p*_1_ is the concentration of the pathway enzymes and v*_ME_* and *κ*_*ME*_ are the turnover rate and Michalis-Menton constants, respectively. The dynamics of the synthetic metabolite are given by

$$\frac{dX}{dt}=\underset{\mathrm{conversion of}\;M\;\mathrm{to}\;X}{\underset{︸}{\left(\frac{v_{ME}\cdot M\cdot p_{1}}{\kappa_{ME}+M}\right)}}-\underset{\mathrm{dilution by growth}}{\underset{︸}{\lambda \cdot X}}$$

### Model Fitting

The model was parametrized using MATLAB’s in-built particle swarm function *particleswarm*, with a large population of 500 individuals, and fitting ceasing after 500 generations.

The model was fit in the exponential growth regime and growth rate was varied by simulating the response of the model to a range of ϕ and k_cm values. Given that the data were on scales of different magnitude, the sum of squared error was normalized by the sum of the respective experimental data squared. The cost function used in the optimization algorithm was made up of a weighted sum of errors from mass fraction data from^22^ and.^34^ We defined the following sum of squared errors for the growth rate λ and ribosomal mass fraction *R* from^22^

$$\sigma_{\lambda ,\mathrm{Scott}}=\frac{\sum \left({\left(\lambda_{sim,\mathrm{Scott}}-\lambda_{\exp,\mathrm{Scott}}\right)}^{2}\right)}{\sum \left({\lambda_{\exp,\mathrm{Scott}}}^{2}\right)}$$

$$\sigma_{R,\mathrm{Scott}}=\frac{\sum \left({\left(R_{sim,\mathrm{Scott}}-R_{\exp,\mathrm{Scott}}\right)}^{2}\right)}{\sum \left({R_{\exp,\mathrm{Scott}}}^{2}\right)}$$

where the subscript *Scott* denotes the data from^22^ and the simulations were carried out for a range of ϕ and *k*_*cm*_ values. We defined the following sum of squared errors for the growth rate λ, ribosomal mass fraction *R* and peptide elongation rate Γ and θ from^34^

$$\sigma_{\lambda ,DB}=\frac{\sum \left({\left(\lambda_{sim,DB}-\lambda_{\exp,DB}\right)}^{2}\right)}{\sum \left({\lambda_{\exp,DB}}^{2}\right)}$$

$$\sigma_{R,DB}=\frac{\sum \left({\left(R_{sim,DB}-R_{\exp,DB}\right)}^{2}\right)}{\sum \left({R_{\exp,DB}}^{2}\right)}$$

$$\sigma_{\Gamma ,DB}=\frac{\sum \left({\left(\Gamma_{sim,DB}-\Gamma_{\exp,DB}\right)}^{2}\right)}{\sum \left({\Gamma_{\exp,DB}}^{2}\right)}$$

$$\sigma_{\theta ,DB}=\frac{\sum \left({\left(\theta_{sim,DB}-\theta_{\exp,DB}\right)}^{2}\right)}{\sum \left({\theta_{\exp,DB}}^{2}\right)}$$

The weighted cost function used for data fitting was:

$$\mathrm{cost}=10\cdot \sigma_{\lambda ,DB}+5\cdot \sigma_{\lambda ,Scott}+5\cdot \sigma_{R,Scott}+\sigma_{R,DB}+\sigma_{\Gamma ,\mathrm{DB}}+\sigma_{\theta ,DB}$$

Parameters estimated from fitting experimental data are shown in Table S2.

### Simulating the Mathematical Modeling

The system of ordinary differential equations was implemented in MATLAB2019b (The MathWorks Inc., Natick, MA, USA) and its behavior simulated using the in-built stiff solver *ode15s*. All simulations were initiated with 100 molecules of each protein species, including ribosomes, and 10^3^ molecules of M, *t*_*u*_ and *t*_*c*_. All simulations were run to steady state by increasing the simulation time span until the maximal absolute value of the derivate was very small (<10^–3^). Isocost lines were simulated by incorporating two additional genes as described above and varying *u*_1_ between 0 and 1 while *u*_2_ was held constant at 1. The parameters used for the simulations are shown in Table S3.

### Genome Scale Metabolic Modeling

Flux balance and flux variability analyses were conducted using the COBRA toolbox (ver3) in MATLAB 2023b. Linear programming problems were solved using the GROUBI solver under an academic license.

## Supplementary Material



## Notes

Supplementary figures including extended computational simulations, experimental results, molecular methods, and additional references (PDF)
