# Supplementary material for: Gene Expression Depends on the Interplay Among Growth, Resource Biogenesis, and Nutrient Quality
Source: ACS Synth Biol. 2025 May 29;14(6):2012–29. doi: 10.1021/acssynbio.4c00828 (PMC12186681; doi:10.1021/acssynbio.4c00828)
Supplement: Supplementary file 1 [file sb4c00828_si_001.pdf]

Supplementary information for 'Gene expression capacity is determined by the interplay between growth rate, resource biogenesis and nutrient quality'

by

Juhyun Kim, Alexander P.S. Darlington, Said Muñoz-Montero, Rafael Molina-Montenegro, Perrine Dalby, Noemí Herrera, Alice Banks, Satya Prakash, Karen Polizzi, Declan G. Bates and José I. Jiménez

[1] School of Life Science, BK21 FOUR KNU Creative BioResearch Group, Kyungpook National University, Daegu, 41566, Republic of Korea

[2] School of Engineering, University of Warwick, Coventry, CV4 7AL, United Kingdom

[3] Department of Life Sciences, Imperial College London, South Kensington Campus, London, SW7 2AZ, United Kingdom

[4] Department of Chemical Engineering, Imperial College London, South Kensington Campus, London, SW7 2AZ, United Kingdom

[†] These authors contributed equally to this work

* Corresponding authors: Alexander P.S. Darlington (a.darlington1@warwick.ac.uk) and José I. Jiménez (j.jimenez@imperial.ac.uk)

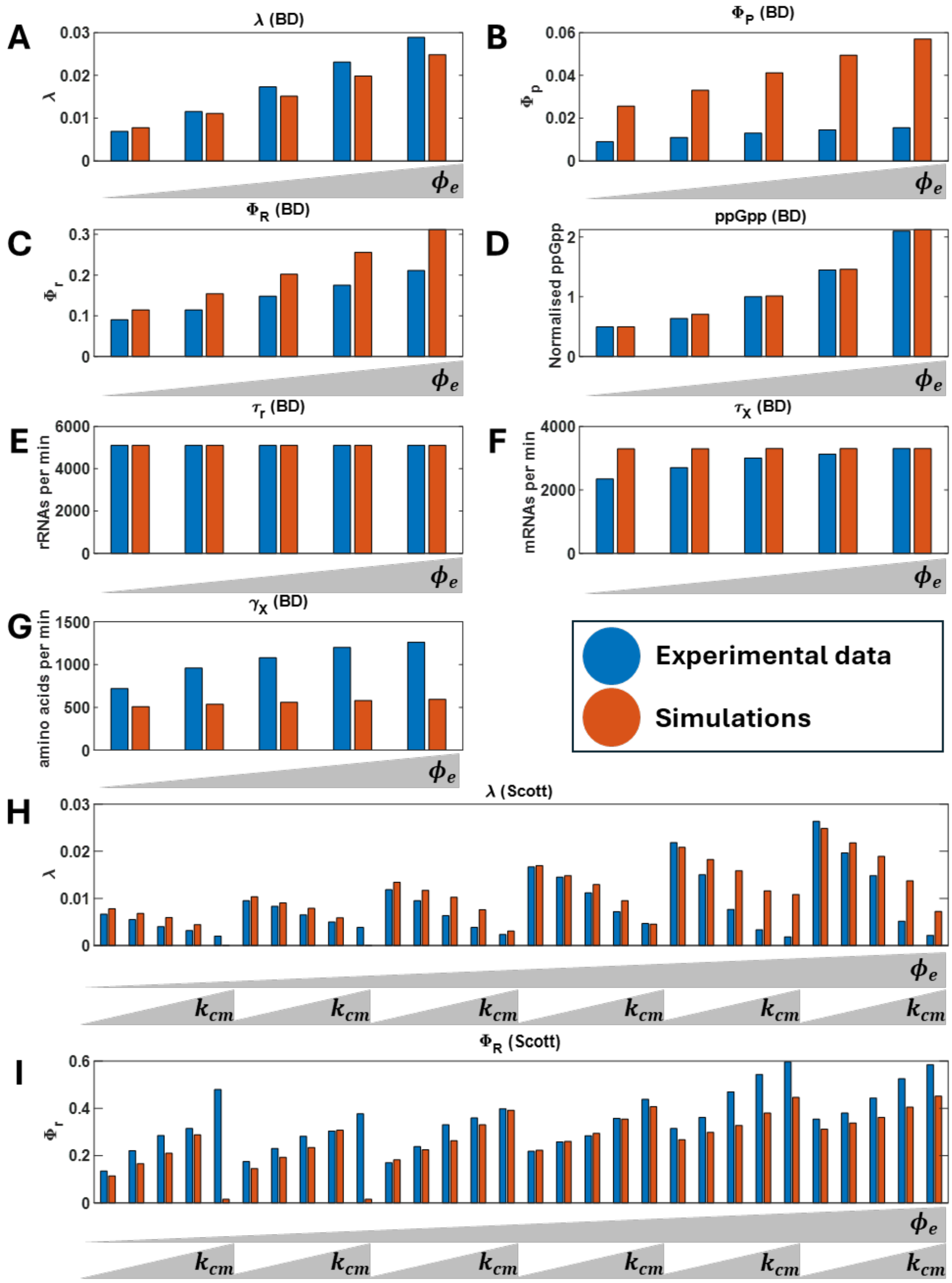

Figure S1. Model data fitting. The model was fit as described in the main text to data from [1] and [2]. ϕ_e represents the range the 'energy threshold' parameter was varied along for the simulations. ϕ_e was varied on a log scale between 0.0424 and 0.2575 (determined during the data fitting regime) and k_{cm} represents translational inhibition (varied according to the original reference [2], with a constant of proportionality of 0.004). The experimental data is shown in blue and the simulation results are shown in orange. **(A)** Mid exponential phase growth rate from [1] (1/min). **(B)** Mid exponential phase RNA polymerase proteome mass fraction from [1]. **(C)** Mid exponential phase ribosome proteome mass fraction from [1]. **(D)** ppGpp concentration from [1] normalised by the mid-value from [1]. **(E)** Mid exponential phase rRNA transcriptional elongation rate from [1]. **(F)** Mid exponential phase mRNA transcriptional elongation rate from [1]. **(G)** Mid exponential phase peptide elongation rate from [1]. **(H)** Growth rate of cells (1/min) subjected to varying carbon source (ϕ_e) and translational inhibition (k_{cm}) from [2]. **(I)** Ribosome mass fraction of cells subjected to varying carbon source (ϕ_e) and translational inhibition (k_{cm}) from [2].

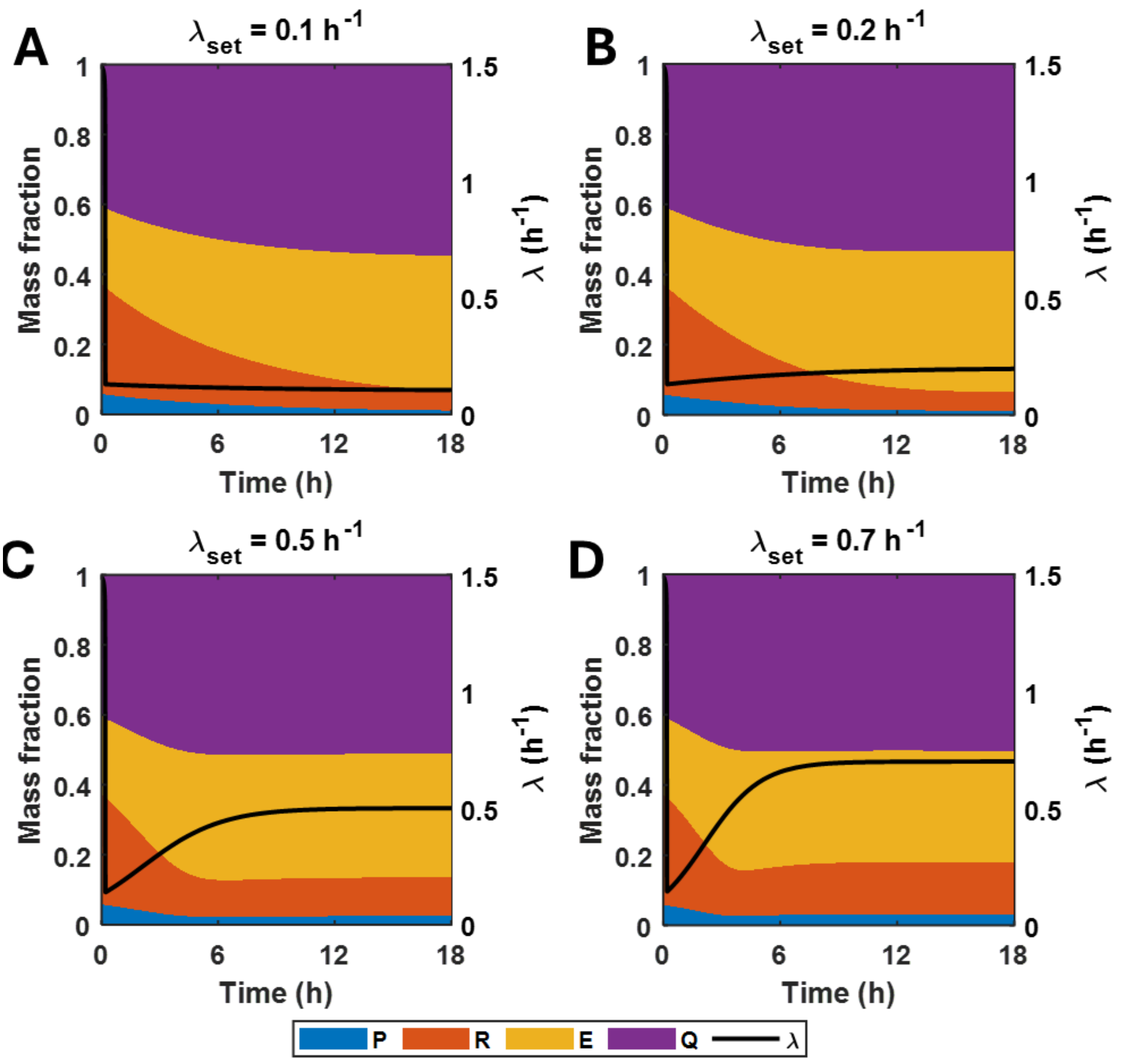

Figure S2. Simulations of the dynamics of the cellular economy in the early phases of chemostat growth. The model (without circuit induction, i.e. $u_1 = u_2 = 0$) was initially simulated in the mid-exponential setting ($dS/dt = dN/dt = 0$, $k_{in} = \delta = 0$) to determine the initial conditions (time $t = 0$ h) for chemostat simulations. To simulate chemostat the model is initialised at the at the previously obtained mid-exponential steady state and $S(0) = 10^8$ molecules, $N(0) = 1$ cells, $k_{in} = 10^6$ S molecules per min and the chemostat dilution rate δ set to λ_{set} (in the units of per minute). Plots show the time evolution of the cellular economy (proteome) and growth rate (λ) from the mid exponential phase initial conditions. The proteome fractions shown are RNA polymerase (P), ribosomes (including nonfunctional r-proteins, free and translating ribosome, R), host enzymes (E) and host biomass proteins from the q-fraction (Q). These simulations of the cellular economy do not include circuit induction (i.e. $u_1 = u_2 = 0$ throughout). **(A)** Simulations with $\delta = 0.1$ h^{-1}. **(B)** Simulations with $\delta = 0.2$ h^{-1}. **(C)** Simulations with $\delta = 0.5$ h^{-1}. **(D)** Simulations with $\delta = 0.7$ h^{-1}.

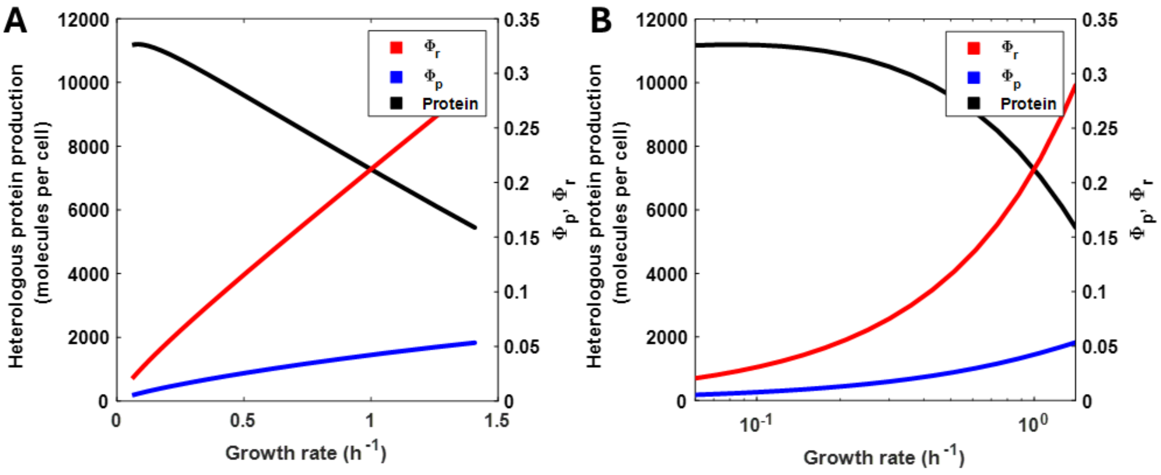

Figure S3. Model predictions of heterologous protein productions and the cellular economy across a range of growth rates. The model was simulated across a range of set dilution rates with the resulting protein production and cellular economy (as Φ_p mass fraction of RNA polymerase, Φ_r mass fraction of ribosomes) shown. To simplify the analysis only a single protein was simulated (i.e. $u_1 = 0$, $u_2 = 1$). **(A)** Protein production and the composition of cellular economy across different chemostat growth rates. **(B)** The same simulation results as in panel (A) but shown on a log scale.

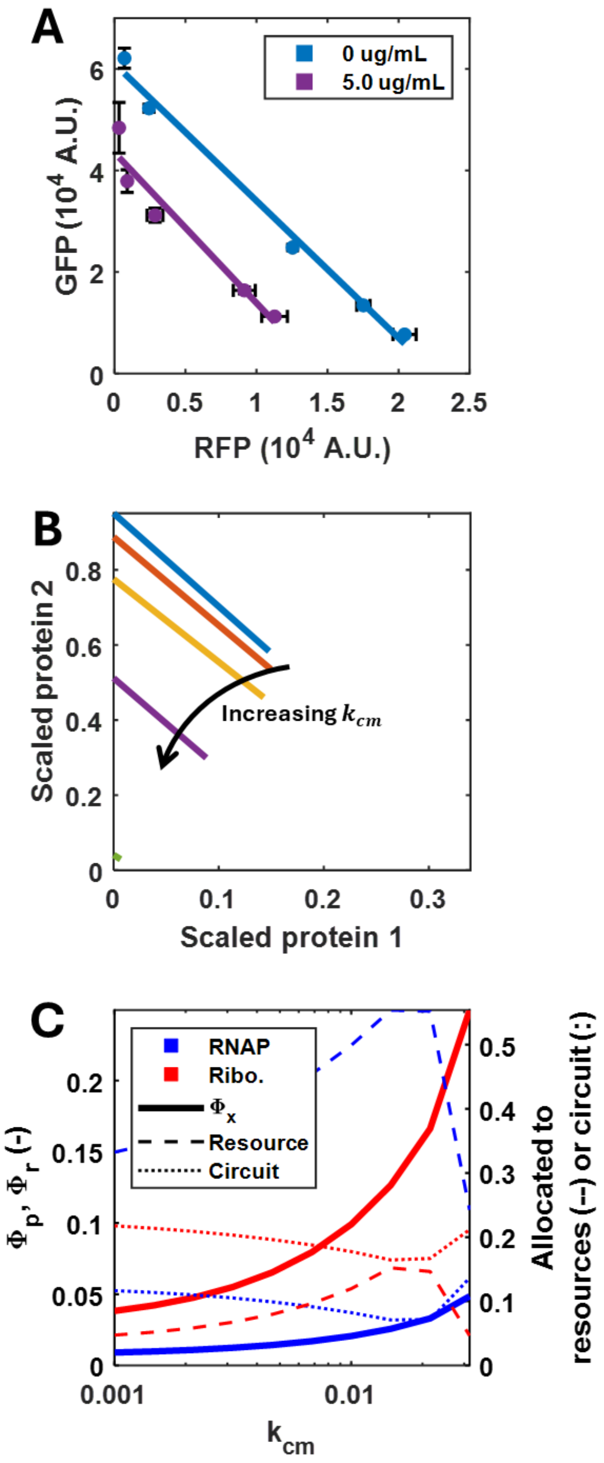

Figure S4. Effect of selective inhibition of translation on the two-reporter circuit gene expression. (A) The strain carrying the GFP and RFP reporters was cultured in the minichemostat in the presence of sublethal concentrations of chloramphenicol. Points represent mean ± 1 SD. N = 3. **(B)** Simulations of the steady state concentration of the two-reporter circuit normalized by maximum protein production, for different antibiotic transcriptional inhibition constant ranging from k_{cm} 0 to 0.1. Isocost lines were simulated by varying u_1 between 0 and 1 while maintaining u_2=1. Simulations were carried out as described in the Methods section. **(C)** Simulations of the cell's internal resource economy expressing the two-reporter circuit (with induction constants of u_1=0, u_2=1) over a range of the RNA polymerase inhibition constant k_{cm} varied on a log10 scale from -3 to -0.05.

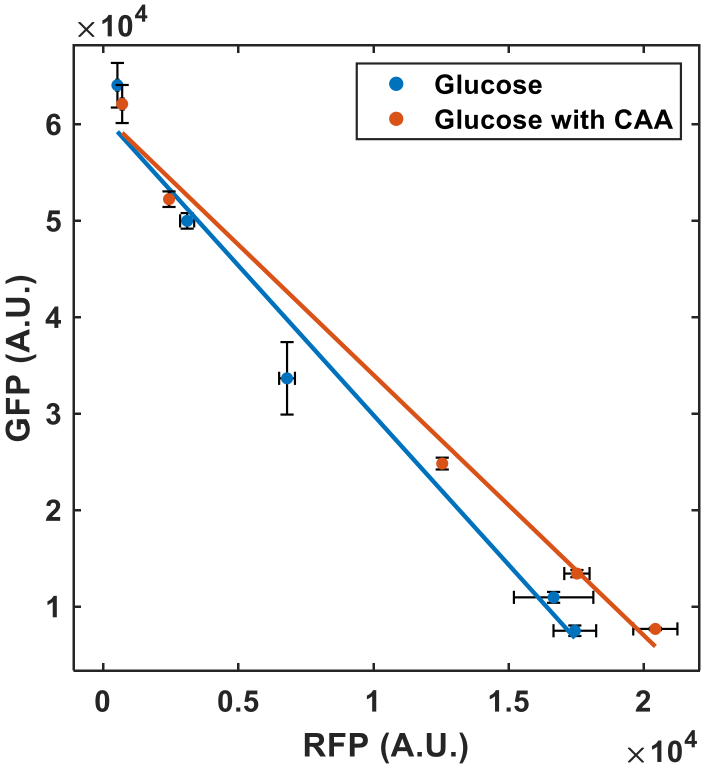

Figure S5. Effect of additional nutrients on the circuit gene expressions. The dual reporter strain was cultured in the continuous system containing M9 minimal medium supplemented with glucose as a sole carbon source. To determine the level of the gene expression coupling, different concentration of AHL applied into each bioreactor and measured both GFP and RFP intensities. The counterpart expression profile from Figure 2A also displayed in this figure. Points represent the mean ± 1 SD. $N = 3$.

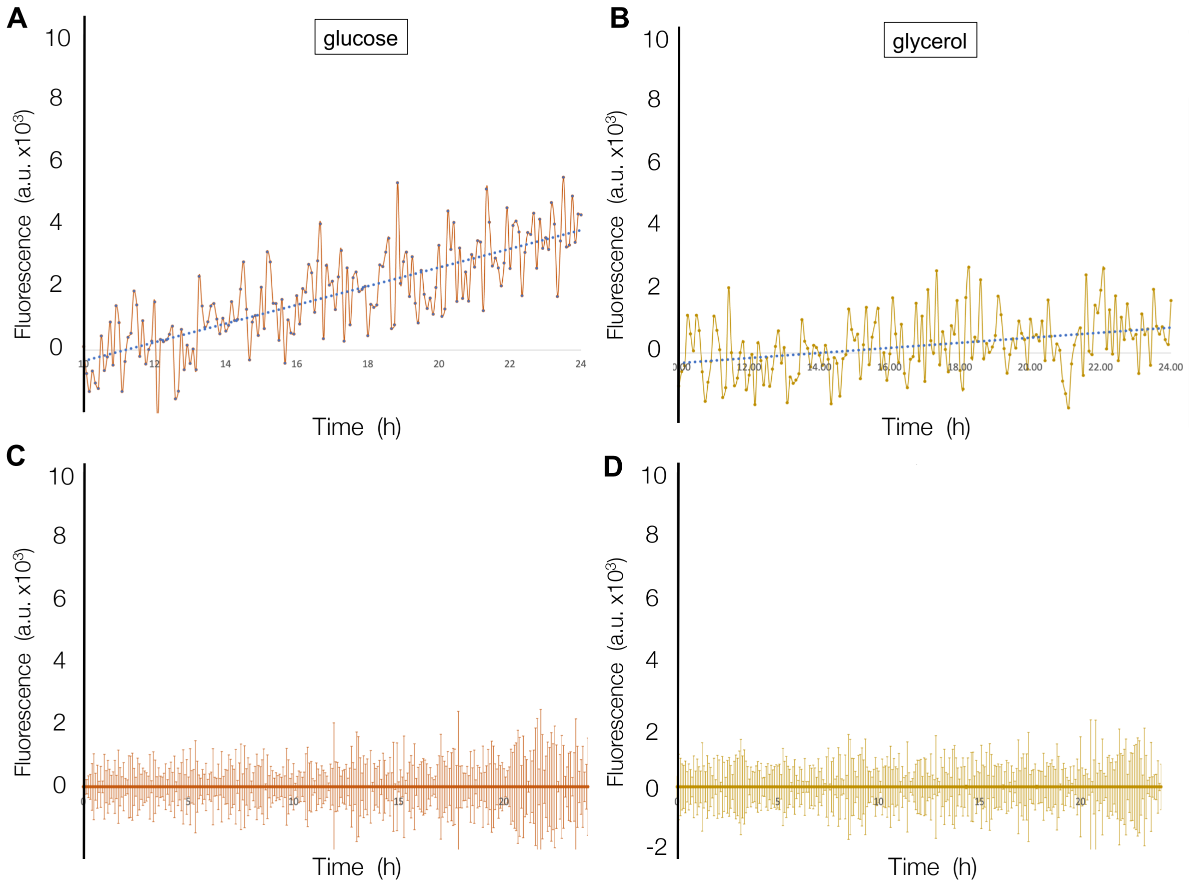

Figure S6. Determination of translation elongation rates. Representative kinetics of mCherry production of in cell-free extracts of cultures grown in glucose (panels A and C) or glycerol (panels B and D). Panels in the upper row (A and B) show a low but detectable increase in fluorescence when 50 nM of an mRNA coding for mCherry were added to the reaction. The lower panels (C and D) are the corresponding negative controls to which no mRNA was added.

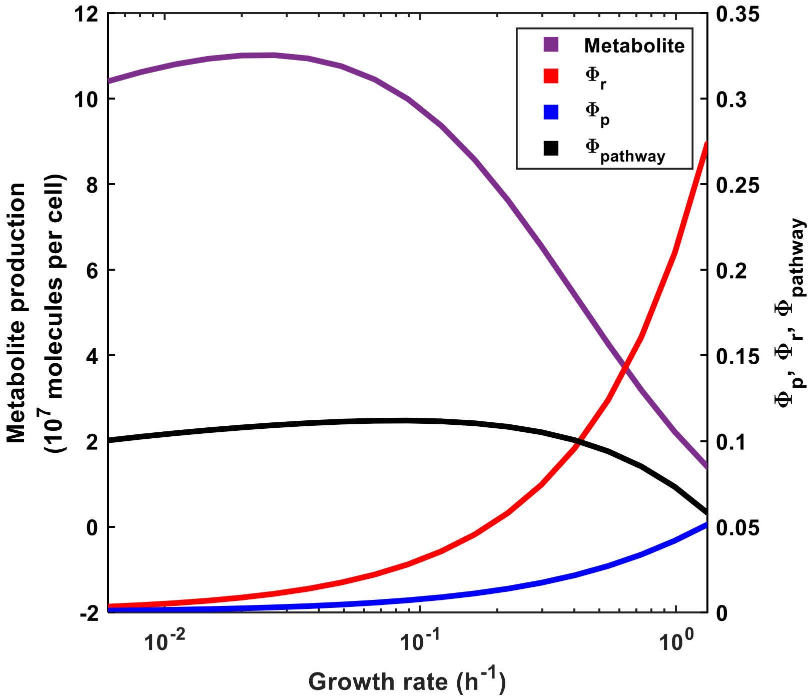

Figure S7. Model predictions of heterologous metabolite productions and the cellular economy across a range of growth rates. The model was simulated across a range of set dilution rates with the resulting metabolite production and cellular economy (as Φ_p mass fraction of RNA polymerase, Φ_r mass fraction of ribosomes, $\Phi_{pathway}$ mass fraction of pathway enzymes) shown.

Table S1. Bacterial strains, plasmids and oligonucleotides used in this study

***E. coli* strains**	**Description**	**Reference**
MG1655	F^-, λ^-, *ilvG*-, *rfb-50*, *rph-1*	[3]
MG1655 L9-*msf*GFP	MG1655 derivative with a C-terminal msf GFP tag in 50S ribosomal protein L9, encoded by *rplI* gene	This study
BW25113	Parental strain for the Keio Collection of single gene knockouts Δ(*araD-araB*)567,Δ*lacZ4787*(::*rrnB-3*), λ-, *rph-1*, Δ(*rhaD-rhaB*)568, *hsdR514*	[4]
BW25113 (Δ relA)	BW25113 derivate with a deletion of *relA* genes	Keio collection
BW25113 (Δ spoT)	BW25113 derivate with a deletion of *spoT* genes	This study
DH5α	Cloning host: F^- Φ80 *lacZ*Δ*M*Δ15 (*lacZYA-argF*), U169, *recA1, endA1, hsdR17, R-M+, supE44, thil, gyrA, relA1*	[5]
DH5α λpir	DH5α λpir phage lysogen	Victor de Lorenzo's collection
SQ37	MG1655 derivate, Δ*rrnE*	[6]
SQ40	MG1655 derivate, Δ*rrnEG*	[6]
SQ49	MG1655 derivate, Δ*rrnGBA*	[6]
SQ53	MG1655 derivate, Δ*rrnGBAD*	[6]
SQ78	MG1655 derivate, Δ*rrnGADE*	[6]
**Plasmid**	**Description**	**Reference**
pEMG	Suicide plasmid, Km^R, oriR6K, *lacZa* with two flanking I-SceI sites	[7]
pEMG-rplI-msfGFP	Same as pEMG but carrying the msf GFP gene with upstream and downstream flanking regions of the C-terminus of *E. coli* rplI gene	This study
pKD4	Template for Km cassette, oriR6Kgamma, *bla, aphA*	[8]
pKD46	Red recombinase expression vector, repA101ts, oriR101, P_{araB} *exo, bet, gam araC bla*	[8]
pSEVA63-Dual	pSEVA631 carrying the circuit MBP 1.0	[9]

PbolA-GFP	Transcriptional fusion between a RpoS responsive promoter and GFP, *kan*	10
**Oligos**	**Sequence (5' --> 3')**	
TS1^{rplI}F	AGGGATAACAGGGTAATCTGCGCTCGCTACCTGTCCCTGCT	
TS1^{rplI}R	TTTACTGCCACCGCCACCGCTTTCAGCTACTACGTTTACGA	
rplI-msfGFP-F	GAAAGCGGTGGCGGTGGCAGTAAAGGTGAAGAACTGTTCACCG	
rplI-msfGFP-R	TACGTCTCGTTGAATAACGAATTATTTGTAGAGTTCATCCAT	
TS2^{rplI}F	CATGGATGAACTCTACAAATAATTCGTTATTCAACGAGACGT	
TS2^{rplI}R	GCCTGCAGGTCGACTCTAGAGTATTTATTGCAAGATGTCGAAT	
spoT KO F	TTACCGCTATTGCTGAAGGTCGTCGTTAATCACAAAGCGGGTCGCCCT TGGTGTAGGCTGGAGCTGCTTC	
spoT KO R	CGTGCATAACGTGTTGGGTTCATAAAACATTAATTTCGGTTTCGGGTG ACATGGGAATTAGCCATGGTCC	

Table S2: Parameter values identified during data fitting

Parameter	Units	L.B.	U.B.	Fit value	Notes
min φ_e	unitless	−3	2	−0.26496	Varied on a log scale 10x, x is fit. $\varphi_e = 0.543$
max φ_e	unitless	−3	2	0.64804	Varied on a log scale 10x, x is fit. $\varphi_e = 4.447$
$\kappa_{r,\tau}$	molecules	0	1000	536.2379	
κ_τ	molecules	0	1000	687.0312	
κ_γ	molecules	0	1000	177.4952	
κ_H	molecules	0	M_0 / n_X	15042.9702	
o_X	molecules	0	1000	99.0688	
o_R	molecules	0	1000	864.5958	
$g_{T/E,0}$	molecules	10	100	14	No. of promoters
k, $g_{H,0}$ scaling factor	unitless	1	10	9.787	$g_{H,0}$ is indirectly set $k \cdot g_{T/E,0} \therefore g_{H,0} = 132$
$g_{P,0}$	molecules	0	100	2	No. of promoters
φ_a	unitless	−3	2	0.98085	Varied on a log scale 10x, x is fit. $\varphi_a = 9.57$
φ_n	unitless	−3	2	1.2961	Varied on a log scale 10x, x is fit. $\varphi_n = 19.77$

Table S3: Known parameters

Parameters	Value	Ref.
φ_X	10	Calculated in [11]
φ_L	6	Calculated in [11]
v_T	726 molecules per min	Reported in [12]
v_E	5800 molecules per min	Reported in [12]
$k_{\{T, E\}}$	1000 molecules	Assumed
$g_{R,0}$	56	Number of r-protein genes in E. coli
$g_{r,0}$	22	Number of rRNA genes in E. coli
q_X	40	Estimation based on data in [1]
q_r	50	Estimation based on data in [1]
$n_{\{T, E, A, H\}}$	330 amino acids	Avg. *E. coli* gene is 1000 nucleotides
n_P	3636 amino acids	Size of the E. coli RNA polymerase core complex
n_R	7459 amino acids	Size of the E. coli ribosome and associated factors
n_r	4566 nucleotides	Length of the in E. coli rRNA operon
β_j	1 cell/(molecule · min)	Assumed as in [12]
μ_j	1 1/min	Assumed as in [12]
b_j	1 cell/(molecule · min)	Assumed as in [12]
u_j	1 1/min	Assumed as in [12]
b_Q	1 cell/(molecule · min)	Assume diffusion limited as in [9]
u_Q	1 1/min	Assume diffusion limited as in [9]
δ_{mj}	0.1 1/min	mRNA's have a half life of minutes[13]
h_H	4	Assumed
$\tau_{r,max}$	5100 nucleotides/min	[1]
$\tau_{X,max}$	3300 nucleotides/min	[1]
γ_{max}	1260 amino acids/min	[1]
M_0	108 amino acids	[13]

Supplementary references

1. Bremer Hans & Dennis Patrick P. Modulation of Chemical Composition and Other Parameters of the Cell at Different Exponential Growth Rates. *EcoSal Plus* **3**, 10.1128/ecosal.5.2.3 (2008).
2. Scott, M., Gunderson, C. W., Mateescu, E. M., Zhang, Z. & Hwa, T. Interdependence of Cell Growth and Gene Expression: Origins and Consequences. *Science* **330**, 1099–1102 (2010).
3. Blattner, F. R. *et al.* The Complete Genome Sequence of Escherichia coli K-12. *Science* **277**, 1453–1462 (1997).
4. Baba, T. *et al.* Construction of Escherichia coli K-12 in-frame, single-gene knockout mutants: the Keio collection. *Mol. Syst. Biol.* **2**, 2006.0008 (2006).
5. Hanahan, D. & Meselson, M. [24] Plasmid screening at high colony density. in *Methods in Enzymology* vol. 100 333–342 (Academic Press, 1983).
6. Quan, S., Skovgaard, O., McLaughlin, R. E., Buurman, E. T. & Squires, C. L. Markerless Escherichia coli rrn Deletion Strains for Genetic Determination of Ribosomal Binding Sites. *G3 GenesGenomesGenetics* **5**, 2555–2557 (2015).
7. Martínez-García, E. & de Lorenzo, V. Engineering multiple genomic deletions in Gram-negative bacteria: analysis of the multi-resistant antibiotic profile of Pseudomonas putida KT2440. *Environ. Microbiol.* **13**, 2702–2716 (2011).
8. Datsenko, K. A. & Wanner, B. L. One-step inactivation of chromosomal genes in Escherichia coli K-12 using PCR products. *Proc. Natl. Acad. Sci.* **97**, 6640–6645 (2000).
9. Darlington, A. P. S., Kim, J., Jiménez, J. I. & Bates, D. G. Dynamic allocation of orthogonal ribosomes facilitates uncoupling of co-expressed genes. *Nat. Commun.* **9**, 695 (2018).
10. Patange, O. *et al.* Escherichia coli can survive stress by noisy growth modulation. *Nat. Commun.* **9**, 5333 (2018).
11. Liao, C., Blanchard, A. E. & Lu, T. An integrative circuit–host modelling framework for predicting synthetic gene network behaviours. *Nat. Microbiol.* **2**, 1658–1666 (2017).

12. Weiße, A. Y., Oyarzún, D. A., Danos, V. & Swain, P. S. Mechanistic links between cellular trade-offs, gene expression, and growth. *Proc. Natl. Acad. Sci.* **112**, E1038–E1047 (2015).

13. Milo, R. & Philips, R. *Cell Biology by the Numbers.* (Garland Science, 2015).
